# Bacterial profile and prevalence of urinary tract infections in pregnant women in Latin America: a systematic review and meta-analysis

**DOI:** 10.1186/s12884-023-06060-z

**Published:** 2023-11-08

**Authors:** Henrique Diório de Souza, Giselle Rodrigues Mota Diório, Stela Verzinhasse Peres, Rossana Pulcineli Vieira Francisco, Marco Aurélio Knippel Galletta

**Affiliations:** 1 Disciplina de Obstetricia, Departamento de Obstetricia E Ginecologia, Faculdade de Medicina FMUSP, Universidade de Sao Paulo, Avenida Doutor Enéas de Carvalho Aguiar, 155 – 10º Andar – Sala 10.037. CEP, São Paulo, BR 05403-000 Brazil; 2 Department of Maternal and Child Health, Medical School of the Federal University of Juiz de Fora, Juiz de Fora, MG Brazil; 3 Department of Internships, Medical School of the Federal University of Juiz de Fora, Juiz de Fora, MG Brazil

**Keywords:** Urinary Tract Infections, Asymptomatic Bacteriuria, Pyelonephritis, Pregnant women, Prevalence, Etiology, Latin America

## Abstract

**Background:**

Given the physiological changes during pregnancy, pregnant women are likely to develop recurrent urinary tract infections (UTIs) and pyelonephritis, which may result in adverse obstetric outcomes, including prematurity and low birth weight preeclampsia. However, data on UTI prevalence and bacterial profile in Latin American pregnant women remain scarce, necessitating the present systematic review to address this issue.

**Methods:**

To identify eligible observational studies published up to September 2022, keywords were systematically searched in Medline/PubMed, Cochrane Library, Embase, Web of Science, and Bireme/Lilacs electronic databases and Google Scholar. The systematic review with meta-analysis followed the Preferred Reporting Items for Systematic Reviews and Meta-Analyses guidelines, and the quality of studies was classified according to the Strengthening the Reporting of Observational Studies in Epidemiology guidelines. The meta-analysis employed a random-effects method with double-arcsine transformation in the R software.

**Results:**

Database and manual searches identified 253,550 citations published until September 2022. Among the identified citations, 67 met the inclusion criteria and were included in the systematic review, corresponding to a sample of 111,249 pregnant women from nine Latin American countries. Among Latin American pregnant women, the prevalence rates of asymptomatic bacteriuria, lower UTI, and pyelonephritis were estimated at 18.45% (95% confidence interval [CI]: 15.45–21.53), 7.54% (95% CI: 4.76–10.87), and 2.34% (95% CI: 0.68–4.85), respectively. Some regional differences were also detected. Among the included studies, *Escherichia coli* (70%) was identified as the most frequently isolated bacterial species, followed by *Klebsiella* sp. (6.8%).

**Conclusion:**

Pregnant women in Latin America exhibit a higher prevalence of bacteriuria, UTI, and pyelonephritis than pregnant women globally. This scenario reinforces the importance of universal screening with urine culture during early prenatal care to ensure improved outcomes. Future investigations should assess the microbial susceptibility profiles of uropathogens isolated from pregnant women in Latin America.

**Trial registration:**

This research was registered at PROSPERO (No. CRD42020212601).

**Supplementary Information:**

The online version contains supplementary material available at 10.1186/s12884-023-06060-z.

## Background

Bacteriuria reportedly affects 1.78–48.3% of pregnant women [1, 2]. Its prevalence depends on the geographic region or age group analyzed. Although the frequency of bacteriuria among pregnant and non-pregnant women appears to be similar, pyelonephritis and recurrent urinary tract infection (UTI) are more frequent in women during the pregnancy-puerperal cycle [3].

UTIs are classified into three subgroups: (a) asymptomatic bacteriuria (ASB); (b) lower UTI, characterized by vaginal mucosa inflammation and irritative urinary tract symptoms; and (c) acute pyelonephritis or upper UTI, a systemic condition. In addition, UTIs can be classified as simple or complicated, depending on the presence of kidney and ureter involvement [4].

Urinary tract dilation and ureteral smooth muscle relaxation during pregnancy increase the susceptibility of the urinary tract to microorganisms. The implementation of universal screening for bacteriuria during pregnancy has substantially reduced the incidence of pyelonephritis; thus, urine culture should be routinely requested for all pregnant women at their first prenatal visit [5–7].

Bacterial colonization of the urinary tract during pregnancy may also be associated with adverse perinatal outcomes such as prematurity [8, 9], low birth weight [10], premature rupture of ovular membranes, and hypertensive syndromes [11–13]. Treating bacteriuria can mitigate some of these adverse obstetric outcomes.

Notably, there are substantial discrepancies in data regarding the prevalence of bacteriuria during pregnancy. In 2019, Latin America recorded the highest regional UTI incidence globally (13,852.9 cases per 100,000 population), the highest mortality from UTI (10.0 per 100,000 population), and the highest number of disability-adjusted life years (DALYs) secondary to UTI (171.3 per 100,000 population) [14]. However, these aspects have been poorly explored in pregnant Latin American women, encouraging the present systematic review with meta-analysis.

The present systematic review would help plan public policies and the implementation of measures to optimize perinatal outcomes related to urinary tract infections during pregnancy.

## Methods

### Study protocol and selection

This systematic review was conducted according to the Preferred Reporting Items for Systematic Reviews and Meta-Analyses. The studies were selected independently by two reviewers (MAKG and HD). Disagreements regarding study inclusion or exclusion were resolved through discussions until a consensus was reached. This systematic review is registered at PROSPERO (No. CRD42020212601).

### Search strategy

The researchers systematically searched Medline/PubMed, Cochrane Library, Embase, Web of Science, and Bireme/Lilacs electronic databases, as well as the Google Scholar search engine. Studies published up to September 2022 were deemed eligible. The studies were searched using the following keywords alone or in combination: bacteriuria OR urinary tract infection OR pyelonephritis OR cystitis OR asymptomatic bacteriuria OR bacteriuria in pregnancy OR urinary tract infection in pregnancy OR pyelonephritis in pregnancy OR cystitis in pregnancy.

### Inclusion and exclusion criteria

Inclusion criteria were as follows: observational studies regarding the prevalence of bacterial urinary tract colonization in pregnant women from Latin American countries; objective diagnostic criteria for UTI, including urine culture reports with minimum bacterial growth of 1 × 10^5^ CFU/ml in a midstream urine sample or of 1 × 10^2^ CFU/ml in a sample obtained by urinary catheterization; published in English, Spanish, or Portuguese; and reported relative risks (RRs) or odds ratios (ORs) or presented original datasets that allowed the calculation of these association measures. This systematic review only included studies conducted in the 20 most populous countries in Latin America, according to the 2020 United Nations Statistical Division: Brazil, Mexico, Colombia, Argentina, Peru, Venezuela, Chile, Guatemala, Ecuador, Bolivia, Haiti, Cuba, Dominican Republic, Honduras, Paraguay, Nicaragua, El Salvador, Costa Rica, Panama, and Uruguay [15]. Exclusion criteria were as follows: non-pregnant women; women residing in non-Latin American countries; incomplete information, such as the absence of prevalence data; duplicate studies; case reports or review articles or secondary analyses; or qualitative studies.

### Data extraction

Two investigators (MAKG and HD) independently extracted relevant data from the studies using a standardized form. The retrieved data included first author details, year of publication, study demographic coverage area, study design, sample size, the prevalence of bacteriuria, the prevalence of UTI, diagnostic criteria for bacteriuria, and association measures such as RRs or ORs. In addition, information on the frequency of microorganism isolation in urine cultures of pregnant women was extracted.

### Quality assessment

Considering the quality, the studies were classified according to the Strengthening the Reporting of Observational Studies in Epidemiology guidelines, analyzing five dimensions: sample population, sample size, percentage of participation among those eligible, result evaluation, and analysis of statistical methods employed. Each of these dimensions received a score ranging from 0 to 2 points. The final total score ranged from 0–10 points, with 10 representing the lowest overall risk of study bias and 0 representing the highest overall risk of study bias [16, 17].

### Statistical analysis

Study-specific synthesized estimates were pooled using the random-effects meta-regression model to estimate the overall prevalence across studies after stabilizing the variance of individual studies using the Freeman-Tukey double-arcsine transformation [18]. Heterogeneity between study results was assessed using Cochran’s Q test and the *I*^2^ index. Publication bias was measured by reviewing the funnel plots and using Begg’s and Egger’s tests. The random-effects model was used to combine highly heterogeneous data. The adjusted ORs and 95%CI of included studies were used for data analysis. Study results were combined to produce a pooled OR-95%CI. Statistical analyses were performed using the R statistical software. Statistical significance was set as *p* < 0.05.

## Results

### Search results

**Fig. 1:**
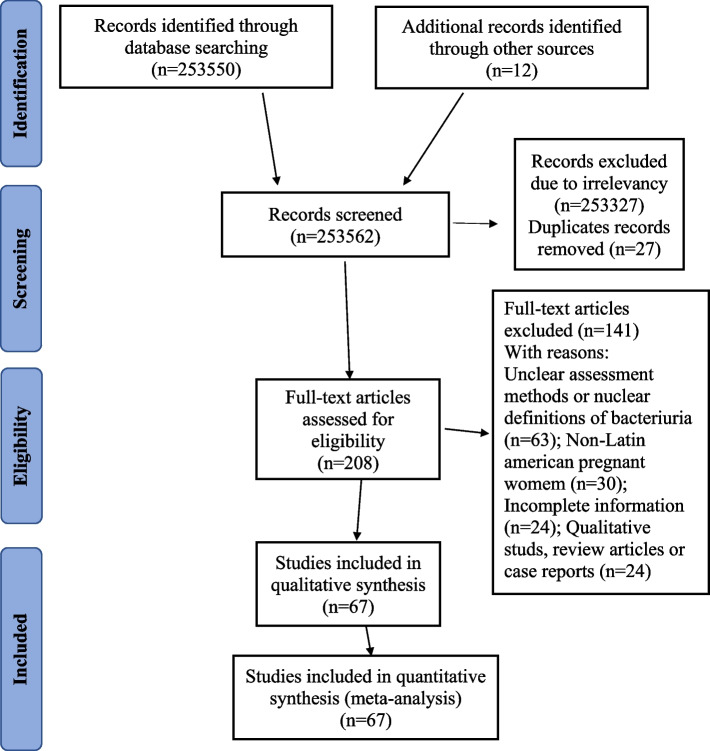
PRISMA flowchart diagram of the study selection

Initial database and manual searches identified 253,550 citations (Medline/PubMed, 267; Google Scholar, 252,446; Lilacs/Bireme, 119; and Embase, 718). Studies were selected by title and abstract, resulting in the exclusion of 253,315 irrelevant studies. Of the remaining 235 citations, 27 were removed as duplicates. Thus, 208 full-text citations were evaluated for eligibility, with 141 excluded owing to unclear assessment methods or uncertain bacteriuria definitions (*n* = 63); non-Latin American pregnant women (*n* = 30); incomplete information (*n* = 24); qualitative studies, review articles, or case reports (*n* = 24). Overall, 67 citations published until September 2022 met the established inclusion criteria and were included in the present systematic review (Fig. 1).

### Study characteristics

The present systematic review with meta-analysis included 67 articles, comprising 111,249 pregnant women from 9 Latin American countries (Brazil, Colombia, Cuba, Ecuador, Guatemala, Paraguay, Peru, Mexico, and Venezuela) (Table 1). All included studies were cross-sectional in design, including 44 published articles, one doctoral dissertation, two master’s theses, and 20 undergraduate course papers. The sample size of the included studies ranged from 34–32,641 pregnant women [19, 20]. The largest number of studies were conducted in Brazil [20], followed by Peru [16] and Mexico [10]. No studies conducted in Argentina, Chile, Bolivia, Haiti, Dominican Republic, Honduras, Nicaragua, El Salvador, Costa Rica, Panama, or Uruguay were selected. The lowest prevalence of bacteriuria was 1.78%, recorded in Mexico, and the highest was 56%, documented in Brazil [1, 19]. Studies reporting the presence of irritative urinary tract symptoms showed that the lowest prevalence of ASB was 1.57% in Ecuador, while the highest was 20.83% in Mexico. The lowest rate of cystitis was 3.1% (Mexico), and the highest was 20.9% (Peru) [21–25].

**Table 1:** Frequency of positive urine cultures and urinary tract infections during prenatal care in pregnant women of all ages in Latin American countries

Authors	Study design	Location	Sample	Year	Bacteriuria in general	ASB^a^	UTI^b^	PYELO^c^
Moran et al. [26]	cross sectional (undergraduate thesis)	Bogotá	1238	1990	***3% (37)***			
Pacheco et al. [27]	cross sectional	Lima	123	1996		***15,4% (19)***		
Ginestre et al. [28]	cross sectional	Zulia	101	2001	***13,86% (14)***			
Cárdenas et al. [29]	cross sectional	Bucaramanga	114	2005		***7,9% (9)***		
Teppa et al. [30]	cross sectional	Caracas	150	2005	***18,7% (28)***			
Blas et al. [31]	cross sectional	Ciudad de México	874	2007		***8,4% (73)***		
Quiroga et al. [23]	cross sectional	Ciudad Obregón	72	2007		***20,83 (15)***	***16,7% (12)***	
Villasante et al. [32]	cross sectional	Lima	300	2007	***17,7% (53)***			
Feitosa et al. [33]	cross sectional	Botucatu	230	2009		***4,3% (10)***	***5,7% (13)***	
Neciosup et al. [34]	cross sectional (undergraduate thesis)	Trujillo	870	2009		***11,8% (103)***		
Pagnonceli et al. [19]	cross sectional	Paraná	34	2010	***56% (19)***			
Pereira et al. [35]	cross sectional	Campo Grande	864	2010		***5,2% (45)***	***7,5% (65)***	***1,2% (10)***
Medic et al. [1]	cross sectional	Puebla	4657	2010	***1,78% (83)***			
Mera et al. [36]	cross sectional (undergraduate thesis)	Chimborazo	140	2010	***24% (33)***			
Darzé et al. [37]	cross sectional	Brotas	260	2011		***12,3% (32)***		
David et al. [38]	cross sectional	Quito	218	2011	***22% (48)***	***12,4% (27)***	***9,6% (21)***	
Giraldo et al. [39]	cross sectional	Natal	94	2012	***29,79% (28)***			
Guerra et al. [40]	cross sectional	Rio de Janeiro	164	2012	***19,5% (32)***			
Mendieta et al. [41]	cross sectional	Cuenca	595	2012		***19,2% (114)***		
Vettore et al. [42]	cross sectional (Phd)	Rio de Janeiro	1091	2013	***45,9% (501)***			
Almeida et al. [43]	cross sectional (MSc)	São Luis	5064	2013	***26,12% (1323)***			
Barros et al. [44]	cross sectional	Recife	124	2013	***37,1% (46)***			
Anduaga et al. [45]	cross sectional	Huatabampo	520	2013	***27,5% (143)***			
Llerena et al. [46]	cross sectional	Ambato	80	2013	***42,5% (34)***			
Vargas et al. [47]	cross sectional	Paucarpata	88	2013	***12,5% (11)***			
Alves et al. [48]	cross sectional	Santa Maria	88	2014	***38,63% (34)***			
Salazar et al. [49]	cross sectional (undergraduate thesis)	Toluca	73	2014	***26,02% (19)***	***16,43% (12)***	***9,58% (7)***	
Castillo et al. [50]	cross sectional (undergraduate thesis)	Piura	19667	2015	***2,48% (488)***			
Fernandez et al. [51]	cross sectional	Lambayeque	47	2015	***21,28% (10)***			
Vega et al. [52]	cross sectional (undergraduate thesis)	Ambato	36	2015	***17% (6)***			
Ramos et al. [53]	cross sectional	Caxias do Sul	432	2016	***12,89% (56)***			
Oliveira et al. [54]	cross sectional	Pará	86	2016	***38,4% (33)***			
Rosado et al. [55]	cross sectional	Tlalnepantla	47	2016	***19,1% (9)***			
Alvarado et al. [56]	cross sectional	Juaréz	145	2016	***13,8% (20)***			
Soloman et al. [57]	cross sectional	Guatemala	210	2016	***9% (19)***			
Mantilla et al. [58]	cross sectional (undergraduate thesis)	Guayaquil	97	2016	***34,02% (33)***			
Macias et al. [22]	cross sectional (undergraduate thesis)	Guayaquil	2041	2016	***7% (150)***	***1,57% (32)***	***4,9% (101)***	***0,8% (17)***
Soria et al. [59]	cross sectional (undergraduate thesis)	Bolivar	729	2016	***24,83% (181)***			
Santos et al. [60]	cross sectional (undergraduate thesis)	Brasília	167	2017	***20,9% (35)***			
Urbina et al. [61]	cross sectional	Barranquilha	226	2017		***10,6% (24)***		
Maquera et al. [62]	cross sectional (undergraduate thesis)	Tacna	164	2017	***9,15% (15)***			
Yaneth et al. [63]	cross sectional (undergraduate thesis)	Trujillo	181	2017	***40,3% (73)***			
Gonzalez et al. [25]	cross sectional (undergraduate thesis)	Abancay	110	2017	***45% (50)***	***15,45% (17)***	***20,9% (23)***	***9,1% (10)***
Melendres et al. [64]	cross sectional (undergraduate thesis)	Ferrenafe	93	2017		***20,4% (19)***		
Santos et al. [65]	cross sectional	Cascavel	798	2018	***15,66% (125)***			
Santos et al. [2]	cross sectional	Maranhão	60	2018	***48,3% (29)***			
Castillo et al. [66]	cross sectional	Lima	1455	2018	***7,4% (108)***			
Sanchez et al. [67]	cross sectional	Huanta	652	2018	***37,1% (242)***			
Fernandez et al. [68]	cross sectional	Las Tunas	1057	2018	***22,51% (238)***			
Pancotto et al. [69]	cross sectional	Veranópolis	538	2019	***25,5% (137)***			
Gonzalez et al. [24]	cross sectional (undergraduate thesis)	Veracruz	954	2019		***13% (124)***	***3,1% (30)***	
Cconisla et al. [70]	cross sectional (undergraduate thesis)	Cusco	239	2019	***14,64% (35)***			
Diaz et al. [71]	cross sectional	Cajamarca	3301	2019	***3,06% (101)***			
Chamoly et al. [72]	cross sectional	Huanchaco	271	2019	***14% (38)***			
Zapana et al. [20]	cross sectional (undergraduate thesis)	Tacna	32641	2019	***29,63% (9673)***			
Placencia et al. [73]	cross sectional (undergraduate thesis)	Cuenca	302	2019	***30,8% (93)***			
Mandujan et al. [74]	cross sectional (undergraduate thesis)	Morelos	525	2020		***13% (70)***		
Fretes et al. [75]	cross sectional	Assunção	202	2020	***2% (5)***			
Cabús et al. [76]	cross sectional (MSc)	Manaus	5925	2021	***22,3% (1322)***			
Rhode et al. [77]	cross sectional	Santa Catarina	164	2021	***14,63% (24)***			
Filho et al. [78]	cross sectional	Distrito Federal	63	2021		***6,3% (4)***		
Macias et al. [79]	cross sectional (undergraduate thesis)	Puebla	10798	2021	***13,28% (1434)***			
Rodriguez et al. [80]	cross sectional	Bucaramanga	838	2021	***14,5% (94)***			
Hoz et al. [81]	cross sectional	Eje cafetero	1131	2021	***14,94% (169)***	***7,69% (87)***	***4,42% (50)***	***2,8% (32)***
Guerra et al. [82]	cross sectional	Villa Clara	3567	2021	***15,9% (568)***			
Planchez et al. [83]	cross sectional	Guanabacoa	129	2021	***40,31% (52)***			
Souza et al. [84]	cross sectional	São Paulo	2935	2021	***11,04% (324)***			

^a^*ASB* asymptomatic bacteriuria

^b^*UTI* urinary tract infection

^c^*PYELO* pyelonephritis

### The overall prevalence of ASB, lower UTI, and pyelonephritis

**Fig. 2:**
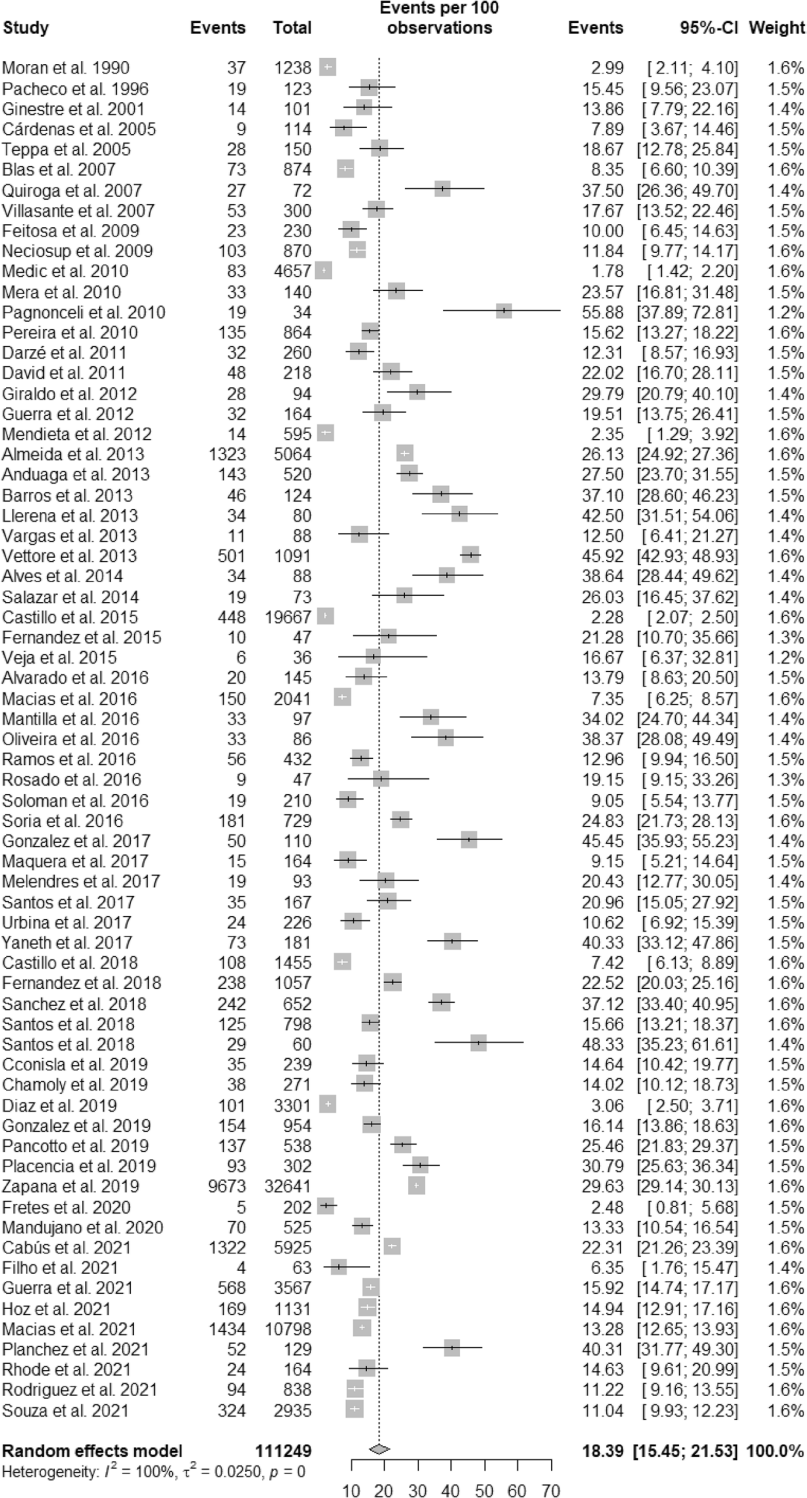
Prevalence of bacteriuria in pregnant women in Latin America

**Fig. 3:**
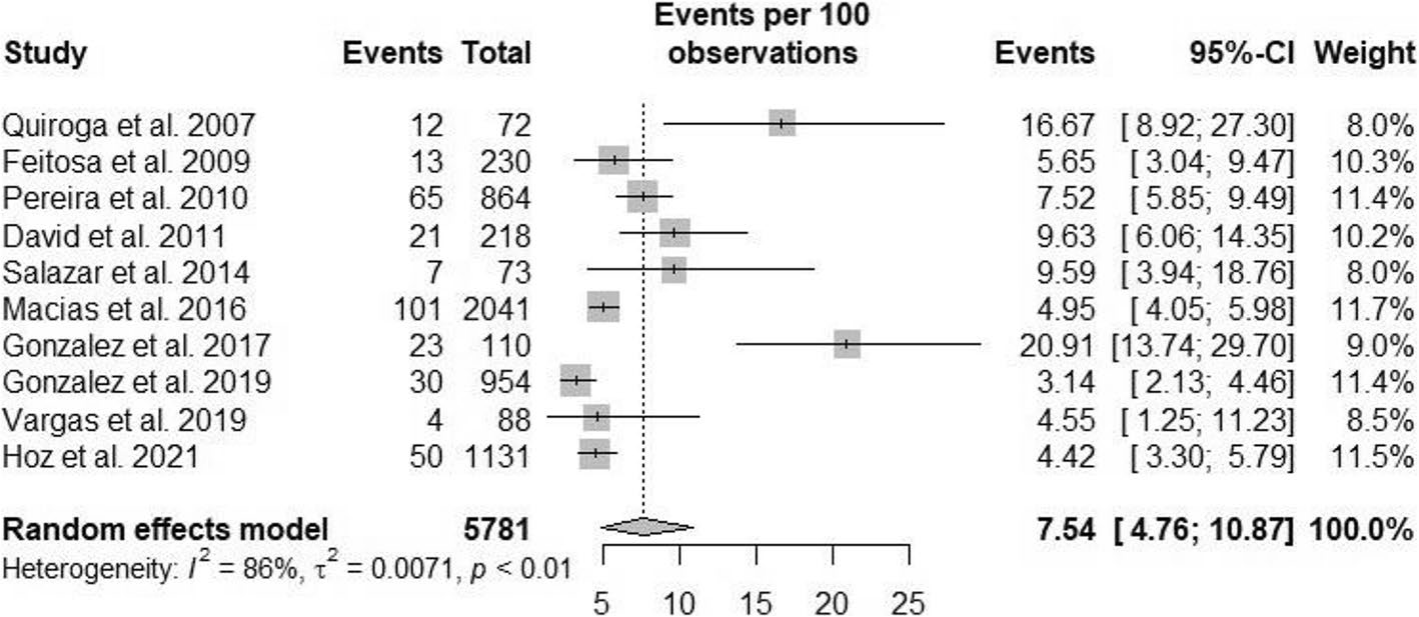
Prevalence of urinary tract infection in pregnant women in Latin America

The heterogeneity rate for ASB prevalence was high (*I*^2^ = 99.5%, *p* < 0.001). The prevalence of ASB in Latin American pregnant women was 18.39% (95% CI: 15.45–21.53) (Figs. 2 and 3) [1, 2, 19–21, 23–31, 34–49, 51–87]. Egger's linear regression test was performed to evaluate the asymmetry of the funnel plot, revealing no statistical significance (*p* = 0.767) (Available in Supplementary Material – Suppl 1).

The heterogeneity rate for lower UTI prevalence was high (*I*^2^ = 86.5%, *p* < 0.001). The prevalence of lower UTI in Latin American pregnant women was 7.54% (95%CI: 4.76–10.87) in 10 studies comprising 5,781 participants [21, 23–25, 35, 38, 47, 49, 81, 86]. Egger’s linear regression test to evaluate the asymmetry of the funnel plot showed statistical significance (*p* = 0.038) (Available in Supplementary Material – Suppl 2).

**Fig. 4:**
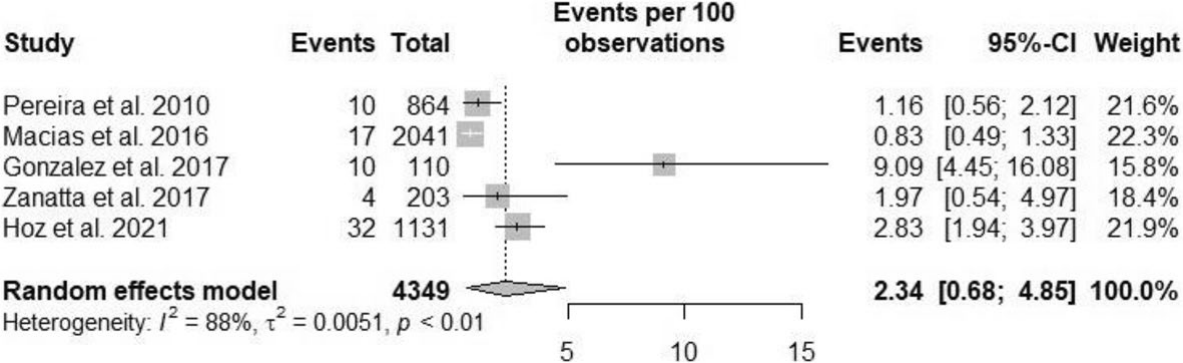
Prevalence of pyelonephritis in pregnant women in Latin America

The heterogeneity rate for the prevalence of pyelonephritis was high (*I*^2^ = 88.4%, *p* < 0.001). The prevalence of pyelonephritis in Latin American pregnant women was 2.34% (95% CI: 0.68–4.85) in five studies comprising a sample size of 4,349 participants (Figs. 4) (Available in Supplementary Material – Suppl 3) [21, 25, 35, 81, 88].

Specific subgroups underwent additional analyses to reduce sample heterogeneity and enhance clinical and public health applicability.

### Subgroup analysis of the prevalence of ASB in articles comprising more than 500 participants

Studies with a larger sample underwent an initial analysis to reduce sample size biases. However, the heterogeneity rate for the prevalence of ASB in Latin American articles with more than 500 participants was also high (*I*^2^ = 99.2%, *p* < 0.001). The prevalence of ASB in Latin American pregnant women was 13.11% (95% CI: 8.42–18.65) in 15 studies comprising 23,782 participants, which was lower than the previous global rate (Fig. 2) (Available in Supplementary Material – Suppl 4) [1, 31, 35, 41, 45, 65–69, 71, 80–82, 84]. Egger’s linear regression test, performed to evaluate the asymmetry of the funnel plot, revealed statistical significance (*p* = 0.02), i.e., persistent publication bias (Supplementary Material – Suppl 5).

### Subgroup analysis of the prevalence of ASB in published Latin American articles, except Brazilian articles

The heterogeneity rate for the prevalence of ASB in the Latin American articles, except the Brazilian articles, was high (*I*^2^ = 98.6%, *p* < 0.05). The prevalence of ASB in Latin American pregnant women was 14.97% (95% CI: 11.10–19.28) in 26 studies comprising 20,896 participants (Supplementary Material – Suppl 6) [1, 27–31, 38, 41, 45–47, 51, 55–57, 61, 66–68, 71, 72, 80–83, 85]. Egger’s linear regression test to evaluate the asymmetry of the funnel plot showed statistical significance (*p* = 0.015).

### Subgroup analysis of the prevalence of ASB in Latin American articles (published or unpublished) with a sample of at least 200 participants, except for Brazilian articles

The heterogeneity rate for the prevalence of ASB in the Latin American articles with a sample of at least 200 participants, except for Brazilian articles, was high (*I*^2^ = 99.8%, *p* < 0.001). The prevalence of ASB in Latin American pregnant women, except Brazilian women, was 12.62% (95% CI: 9.26–16.40) (Supplementary Material – Suppl 7) [1, 20, 21, 24, 26, 29–31, 34, 38, 41, 45–47, 51, 55–57, 59, 61, 66–68, 70–75, 79–83, 85, 87, 89]. Egger’s linear regression test to evaluate the asymmetry of the funnel plot showed statistical significance (*p* = 0.015) (Supplementary Material – Suppl 8).

### Subgroup analysis of the prevalence of ASB considering only Brazilian articles (published or unpublished)

**Fig. 5:**
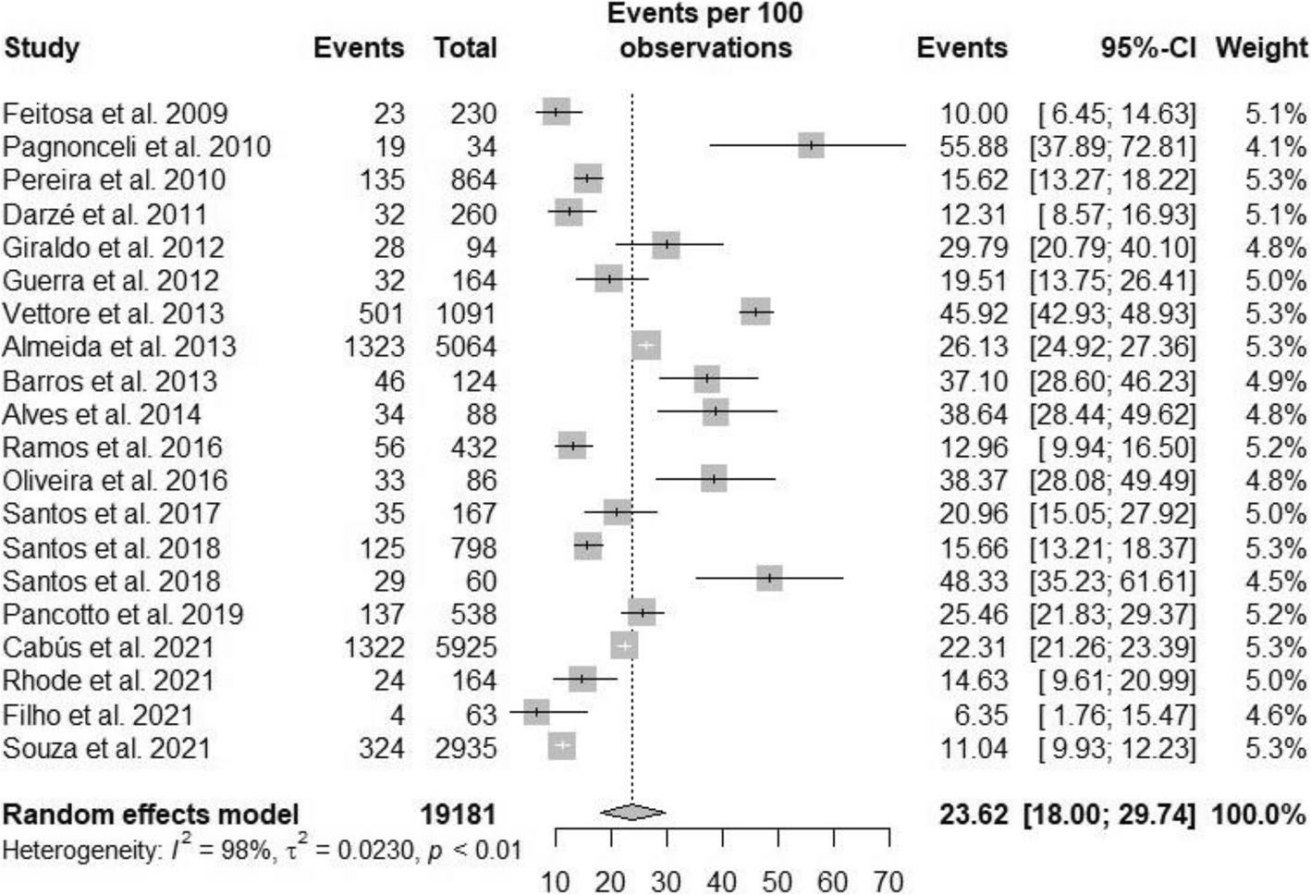
Prevalence of bacteriuria in Brazilian pregnant women, considering published or unpublished studies

Considering only Brazilian articles, the heterogeneity rate for the prevalence of ASB was high (*I*^2^ = 97.5%, *p* < 0.001). The prevalence of ASB in Brazilian pregnant women was 23.62% (95% CI: 18.0–29.74) (Figs. 4 and 5) [2, 19, 23, 35, 37, 39, 40, 42–44, 48, 53, 54, 60, 65, 69, 76–78, 84, 86]. Egger’s linear regression test to evaluate the asymmetry of the funnel plot showed no statistical significance (*p* = 0.831) (Supplementary Material – Suppl 9).

### Subgroup analysis of the prevalence of ASB considering only Brazilian articles (published or unpublished) with a sample of at least 200 participants

Considering only Brazilian articles (published or unpublished), the heterogeneity rate for the prevalence of ASB was high (*I*^2^ = 98.7%, *p* < 0.001). The prevalence of ASB in Brazilian pregnant women was 19.05% (95% CI: 13.18–25.70) in 10 studies comprising 18,137 participants (Supplementary Material – Suppl 10) [2, 35, 37, 42, 43, 53, 65, 69, 76, 84, 86, 90]. Egger’s linear regression test to evaluate the asymmetry of the funnel plot showed no statistical significance (*p* = 0.595) (Supplementary Material – Suppl 11).

### Isolated bacteria

In the present systematic review with meta-analysis of 67 studies, we examined the profile of microorganisms isolated in positive urine cultures of pregnant women residing in the 20 most populous countries in Latin America, comprising a sample of 8,840 urine cultures (Table 2). The most frequently isolated bacterial species in Latin American pregnant women were *Escherichia coli* (pooled prevalence of 70%, 95% CI: 65.3–74.6%); *Klebsiella* sp. (pooled prevalence of 6.4%, 95% CI: 4.3–8.7%); *Staphylococcus* sp., excluding *Staphylococcus aureus*, (pooled prevalence of 3.0%, 95%CI: 1.7%–4.5%); *Proteus mirabilis* (pooled prevalence of 2.8%, 95% CI: 1.9–3.9%); and *Enterobacter* sp. (pooled prevalence of 1.6%, 95% CI: 0.7–2.7%) (Supplementary Material – Suppl 12–21).

**Table 2:** Meta-analyses of the bacterial profile of positive urine cultures of Latin American pregnant women

Bacteria	Sample	Events	Pooled prevalence (95%CI)	*I*^2^ (*p*-value)	Egger (*p*)
***Escherichia coli*** [23, 24, 26–34, 36–38, 40, 46, 47, 50–52, 54–56, 58, 61, 63, 64, 66, 69, 70, 72, 74, 75, 77, 80–84, 88–124]	8840	5974	70.0 (65.3–74.6)	96% (< 0.01)	0.907
***Klebsiella sp.*** [23, 24, 26–34, 36–38, 40, 46, 47, 51, 52, 54, 55, 58, 61, 63, 64, 66, 69, 70, 72, 74, 75, 77, 80–84, 87–121, 123, 124]	8709	694	6.4 (4.3–8.7)	92% (< 0.01)	0.181
***Other Staphylococcus* sp.* (except Staphylococcus aureus)*** [23, 27–30, 32–34, 36–38, 40, 46, 47, 51, 52, 54, 55, 58, 61, 63, 64, 66, 69, 70, 72, 74, 75, 77, 81–84, 88, 90, 92–97, 99, 100, 103–105, 107–121, 123, 124]	7352	288	3.0 (1.7–4.5)	87% (< 0.01)	0.008
***Proteus mirabilis*** [23, 24, 26–34, 36–38, 46, 47, 51, 52, 54, 55, 58, 61, 63, 64, 66, 69, 70, 72, 74, 75, 77, 80–84, 87–97, 99–105, 107–121, 123, 124]	8586	315	2.8 (1.9–3.9)	77% (< 0.01)	0.024
***Enterobacter* sp.** [23, 26–30, 32–34, 36–38, 40, 46, 47, 51, 52, 55, 58, 61, 63, 64, 66, 69, 70, 72, 74, 75, 77, 80–84, 87, 88, 90–100, 102–105, 107–118, 120, 121, 123, 124]	7383	380	1.6 (0.7–2.7)	90% (< 0.01)	0.120
***Enterococcus* sp.** [23, 27–30, 32–34, 36–38, 46, 47, 51, 52, 55, 58, 61, 63, 64, 66, 69, 70, 72, 74, 75, 77, 80–84, 88, 90–97, 99, 100, 103–105, 107–118, 120–124]	7554	395	1.4 (0.5–2.5)	89% (< 0.01)	0.015
***Streptococcus agalactiae*** [23, 27–30, 32–34, 36–38, 40, 46, 47, 51, 52, 55, 58, 61, 63, 64, 66, 69, 70, 72, 74, 75, 77, 81–84, 88, 90–92, 94–97, 99, 100, 103–105, 107–118, 120–124]	6351	233	1.3 (0.5–2.4)	84% (< 0.01)	0.625
***Staphylococcus aureus*** [23, 26–30, 32–34, 36, 38, 46, 47, 51, 52, 55, 58, 61, 63, 64, 66, 69, 70, 72, 74, 75, 77, 81–84, 90, 92–94, 96, 97, 99, 100, 103–105, 107–118, 120, 121, 123, 124]	5728	100	0.4 (0.1–1.0)	63% (< 0.01)	0.471
***Citrobacter sp.*** [23, 27–30, 32–34, 36, 38, 46, 47, 51, 52, 55, 58, 61, 63, 64, 66, 69, 70, 72, 74, 75, 77, 81–84, 87, 90, 94–97, 99, 100, 102–105, 107–118, 120, 121, 123, 124]	6735	41	0.0 (0.0–0.1)	26% (0.04)	0.017
***Pseudomonas aeruginosa*** [23, 27–30, 32–34, 36, 38, 46, 47, 51, 52, 54, 55, 58, 61, 63, 64, 66, 69, 70, 72, 74, 75, 77, 80–84, 88, 90, 92, 94, 96, 97, 99–101, 103–105, 107–118, 120, 121, 123, 124]	5940	21	0.0 (0.0–0.0)	0% (0.68)	< 0.001

## Discussion

Based on the present meta-analysis, the frequency of ASB in Latin American pregnant women was 18.39% (95% CI: 15.45–21.53). This prevalence is higher than frequencies reported in international meta-analyses, including those from Ethiopia (15.37%), Africa (11.1%), and Iran (8.7%) [125–127].

Despite the current propensity to prevent unnecessary antibiotic use, screening and treatment for asymptomatic bacteriuria have become routine in almost all prenatal care guidelines. This occurs because, when the incidence of bacteriuria reaches values ​​greater than 2%, the cost-effectiveness of universal screening appears to be adequate to prevent the occurrence of pyelonephritis during pregnancy [128, 129]. Our study demonstrated a high prevalence of bacteriuria among pregnant Latin American women, reinforcing the importance of universal screening for bacterial colonization of the urinary tract in this population.

In a broad worldwide study in 2019, Tropical Latin America had the highest worldwide UTI incidence standardized by age, with approximately 13,852.9 cases per 100,000 population. Notably, Ecuador presented the highest incidence of UTI globally, with approximately 15,511.3 cases per 100,000 population. In 2019, a global analysis of UTI revealed that the highest mortality rate was recorded in southern Latin America (10 deaths per 100,000 population), and the highest number of DALYs lost was recorded in Tropical Latin America (171.3 per 100,000 population) [14]. Evaluating women only, the highest regional incidences are found, in descending order, in Andean Latin America, Tropical Latin America, Australasia, the Caribbean, and southern Latin America. In 2019, over 404 million individuals had UTIs, with over 236,000 UTI-related deaths recorded [14].

Between 1990 and 2019, the global UTI incidence rate adjusted for age increased from 4,715 to 5,229 per 100,000 population, with the global death rate due to UTI increasing from 1.8 to 3.1 per 100,000 population. A comparison between three-decade-old and current data revealed an absolute increase of approximately 130,000 UTI-related deaths. Over the past three decades, the largest estimated annual percentage changes in UTI incidence rates were observed in Central Latin America (0.48, 95% CI: 0.29–0.67) and Andean Latin America (0.45, 95% CI: 0.4–0.51), and the highest estimated annual percentage changes in UTI mortality rates were documented in southern Latin America (4.92, 95% CI: 4.26–5.59) and Tropical Latin America (3.50, 95% CI: 3.14–3.87). Given the impact of bacterial urinary tract colonization on public health outcomes and the highest global percentage of bacteriuria prevalence documented in Latin America, it is crucial to further explore this topic [14, 130].

Bacteriuria is associated with some adverse perinatal outcomes. Antimicrobial treatment of bacteriuria can reduce the incidence of pyelonephritis in pregnant women (RR 0.24, 95% CI = 0.13–0.41; 12 studies, 2017 women), premature birth (RR 0.34, 95% CI = 0.13–0.88; 3 studies, 327 women) and low birth weight (RR 0.64, 95% CI = 0.45–0.93; 6 studies, 1437 newborns) [131]. There is also evidence that urinary tract infection during pregnancy corresponds to a risk factor for the occurrence of pre-eclampsia (OR 1.31; 95% CI = 1.22–1.40) [13]. The increase in the global mortality rate from UTI in the last three decades, associated with unfavorable obstetric results related to the diagnosis of bacteriuria, reinforces the importance of our study.

Based on the present study, *E. coli* was the most frequently isolated uropathogen in the urine cultures of Latin American pregnant women. The results of this meta-analysis corroborate documented findings in the literature, with up to a 95% frequency of *E. coli* noted among the total number of bacteria isolated from the urinary tract [3].

Considering the total number of uropathogens, the second most isolated bacterial species belonged to the Enterobacteriaceae family (*Klebsiella* sp. or *Proteus* sp.) [3]. The present review revealed that *Klebsiella* sp. was the second most frequently isolated bacterial species among Latin American pregnant women (pooled prevalence of 6.4%, 95% CI: 4.3–8.7), followed by *Proteus mirabilis* as the fourth most frequently identified species in urine cultures (pooled prevalence of 2.8%, 95% CI: 1.9–3.9) and *Enterobacter* sp. as the fifth (pooled prevalence of 1.6%, 95% CI: 0.7–1.7).

This meta-analysis supports previously reported findings regarding the frequency of *Streptococcus agalactiae* among the total number of uropathogens. Collin et al. have analyzed the prevalence of Lancefield group B *Streptococcus* in non-invasive bacterial infections worldwide. The authors identified UTI prevalence rates of 1.61% among bacterial isolates collected from the community and 0.72% among UTI bacterial isolates collected from a hospital environment [132].

Although the present systematic review with meta-analysis presents up-to-date evidence on the prevalence of bacteriuria in Latin American pregnant women, the limitations should be addressed. First, the lack of studies in southern Latin America and Central America may hinder generalization, warranting further investigation of UTIs in these regions. Second, there was significant heterogeneity in the overall pooled prevalence analysis of bacteriuria in Latin American pregnant women, a characteristic maintained in almost all subgroup analyses. Third, we noted a significant publication bias in the general assessment of the prevalence of bacteriuria among pregnant women, both in funnel plots and Egger’s test, reinforcing the need for careful data interpretation. The inclusion of non-published studies in the sub-analyses helped reduce this bias.

Our systematic review with meta-analysis included a total of 67 studies. Of this total research, more than a third had not been published. Of the articles published, only a few were selected in journals indexed in the main international databases. Although bacteriuria is a common topic in obstetric clinical practice, available data on Latin American pregnant women were scarce or difficult to obtain and, according to our review, at rates much higher than those from other regions and indicated by other previous studies, strengthening the value of our current research.

In our study we also examined the profile of microorganisms isolated in positive urine cultures from pregnant women living in the 20 most populous countries in Latin America. This information can help in the construction of care protocols guided by the local bacterial profile, favoring treatments with lower-cost antimicrobials. There are still limitations to Latin American pregnant women's access to health services. In the most populous country in the region, Brazil, in 2021, only two thirds (76.55%) of women had access to adequate prenatal care, that is, starting in the first trimester of pregnancy and with at least six outpatient consultations [133]. Therefore, considering the deficiencies in access to health professionals and laboratory tests during pregnancy, knowledge of the bacterial colonization profile of pregnant women in Latin America can help in planning care for this population.

## Conclusion

UTI and asymptomatic bacteriuria are markedly common among Latin American pregnant women. The prevalence of bacteriuria among Brazilian pregnant women tends to be higher than the mean of Latin America or other regions worldwide. These results reinforce the need for universal screening with urine culture during early prenatal care. Evidence supporting repeated screening for bacteriuria during different trimesters or gestational ages is lacking. Among Latin American pregnant women, the most common microorganism in the etiology of bacteriuria was *E.coli*. Another frequently isolated uropathogen was *S. agalactiae*, with a higher prevalence than that reported in other international studies. This information is highly relevant, as maternal colonization with Lancefield group B streptococci has been associated with adverse perinatal outcomes, such as neonatal sepsis. Given the higher frequency of UTI among Latin American pregnant women, additional studies are needed to assess the effectiveness of screening protocols and better identify the different microbial sensitivity profiles of uropathogens isolated from these women.

### Supplementary Information

**Additional file 1:** **Supp 1. **Funnel plot to test the publication bias in 67 studies with 95% Confidence limits. **Suppl 2.** Funnel plot to test the publication bias in 10 studies with 95% Confidence limits. **Supp 3.** Funnel plot to test the publication bias in 5 studies with 95% Confidence limits. **Supp 4. **Prevalence of bacteriuria in pregnant women in Latin America, considering only articles published with samples greater than 500 individuals. **Supp 5. **Funnel plot to test the publication bias in 15 studies with 95% Confidence limits. **Supp 6.** Prevalence of bacteriuria in pregnant women in Latin America, with the exception of Brazilian articles. **Supp 7.** Prevalence of bacteriuria in pregnant women in Latin America, with the exception of Brazilian articles, in studies with samples greater than 200 individuals. **Supp 8.** Funnel plot to test the publication bias in 28 studies with 95% Confidence limits. **Supp 9.** Funnel plot to test the publication bias in 20 studies with 95% Confidence limits. **Supp 10.** Prevalence of bacteriuria in Brazilian pregnant women, considering published or unpublished studies, in studies with samples greater than 200 individuals. **Supp 11** Funnel plot to test the publication bias in 10 studies with 95% Confidence limits. **Supp 12.** Prevalence of *Escherichia coli* among the total number of uropathogens isolated from urine cultures of Latin American pregnant women. **Supp 13.** Prevalence of *Klebsiella sp*. among the total number of uropathogens isolated from urine cultures of Latin American pregnant women. **Supp 14.** Prevalence of *Staphilococcus sp.* (except*Staphilococcus aureus*) among the total number of uropathogens isolated from urine cultures of Latin American pregnant women. **Supp 15.** Prevalence of *Proteus mirabilis* among the total number of uropathogens isolated from urine cultures of Latin American pregnant women. **Supp 16.** Prevalence of *Enterobacter sp*. among the total number of uropathogens isolated from urine cultures of Latin American pregnant women. **Supp 17.** Prevalence of *Enterococcus sp*. among the total number of uropathogens isolated from urine cultures of Latin American pregnant women. **Supp 18.** Prevalence of *Streptococcus agalactiae* among the total number of uropathogens isolated from urine cultures of Latin American pregnant women. **Supp 19.** Prevalence of *Staphilococcus aureus* among the total number of uropathogens isolated from urine cultures of Latin American pregnant women. **Supp 20** Prevalence of *Citrobacter sp.* among the total number of uropathogens isolated from urine cultures of Latin American pregnant women. **Supp 21.** Prevalence of *Pseudomonas aeruginosa* among the total number of uropathogens isolated from urine cultures of Latin American pregnant women.

## Data Availability

All data generated or analysed during this study are included in this published article [and its supplementary information files].

## Abbreviations

ASB: Asymptomatic bacteriuria
DALY: Disability-adjusted life years
OR: Odds ratios
PRISMA: Preferred Reporting Items for Systematic Reviews and Meta-Analyses
RR: Relative risks
UTI: Urinary tract infection
