# Supplementary material for: Bacterial profile and prevalence of urinary tract infections in pregnant women in Latin America: a systematic review and meta-analysis
Source: BMC Pregnancy Childbirth. 2023 Nov 8;23:774. doi: 10.1186/s12884-023-06060-z (PMC10631168; doi:10.1186/s12884-023-06060-z)
Supplement: Supplementary file 1 — Additional file 1: Supp 1. Funnel plot to test the publication bias in 67 studies with 95% Confidence limits. Suppl 2. Funnel plot to test the publication bias in 10 studies with 95% Confidence limits. Supp 3. Funnel plot to test the publication bias in 5 studies with 95% Confidence limits. Supp 4. Prevalence of bacteriuria in pregnant women in Latin America, considering only articles published with samples greater than 500 individuals. Supp 5. Funnel plot to test the publication bias in 15 studies with 95% Confidence limits. Supp 6. Prevalence of bacteriuria in pregnant women in Latin America, with the exception of Brazilian articles. Supp 7. Prevalence of bacteriuria in pregnant women in Latin America, with the exception of Brazilian articles, in studies with samples greater than 200 individuals. Supp 8. Funnel plot to test the publication bias in 28 studies with 95% Confidence limits. Supp 9. Funnel plot to test the publication bias in 20 studies with 95% Confidence limits. Supp 10. Prevalence of bacteriuria in Brazilian pregnant women, considering published or unpublished studies, in studies with samples greater than 200 individuals. Supp 11 Funnel plot to test the publication bias in 10 studies with 95% Confidence limits. Supp 12. Prevalence of Escherichia coli among the total number of uropathogens isolated from urine cultures of Latin American pregnant women. Supp 13. Prevalence of Klebsiella sp. among the total number of uropathogens isolated from urine cultures of Latin American pregnant women. Supp 14. Prevalence of Staphilococcus sp. (exceptStaphilococcus aureus) among the total number of uropathogens isolated from urine cultures of Latin American pregnant women. Supp 15. Prevalence of Proteus mirabilis among the total number of uropathogens isolated from urine cultures of Latin American pregnant women. Supp 16. Prevalence of Enterobacter sp. among the total number of uropathogens isolated from urine cultures of Latin American pregnant women. Supp 17. Pre [file 12884_2023_6060_MOESM1_ESM.docx]

**Supplementary Files**

**Bacterial Profile and prevalence of urinary tract infections in pregnant women in Latin America: A systematic review and meta-analysis**


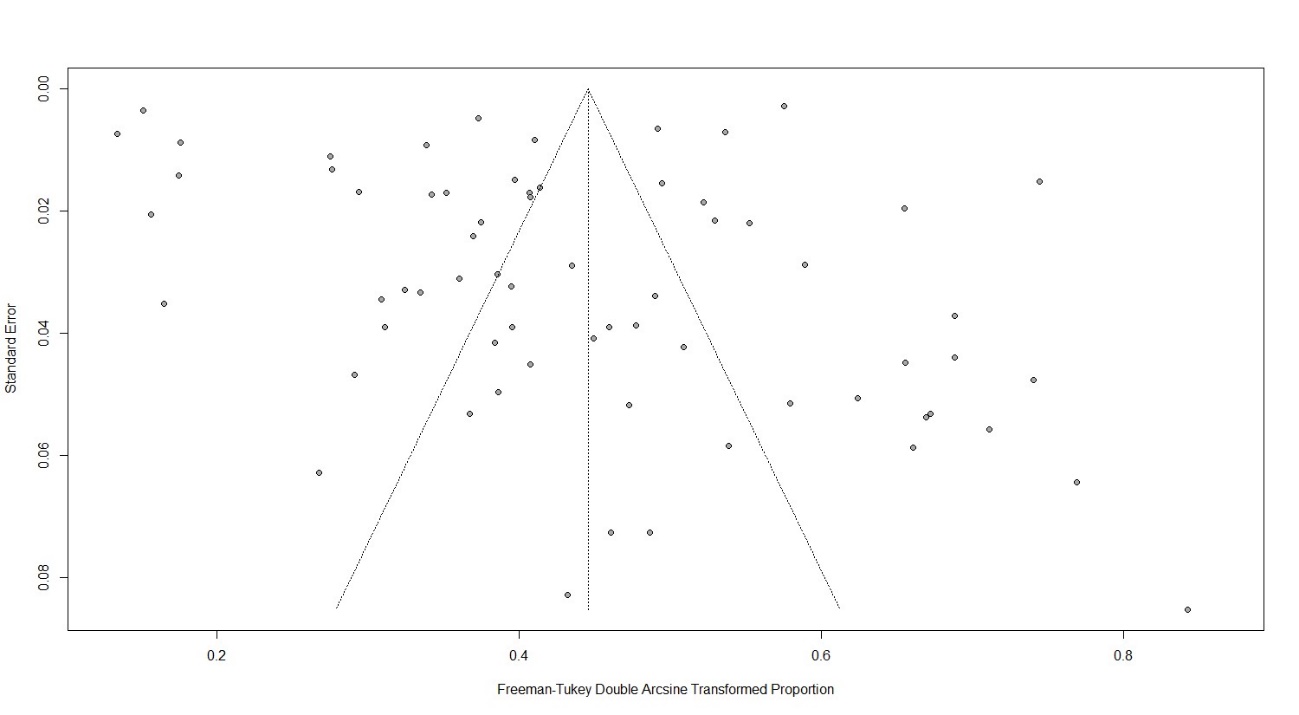


**Supp 1** Funnel plot to test the publication bias in 67 studies with 95% Confidence limits


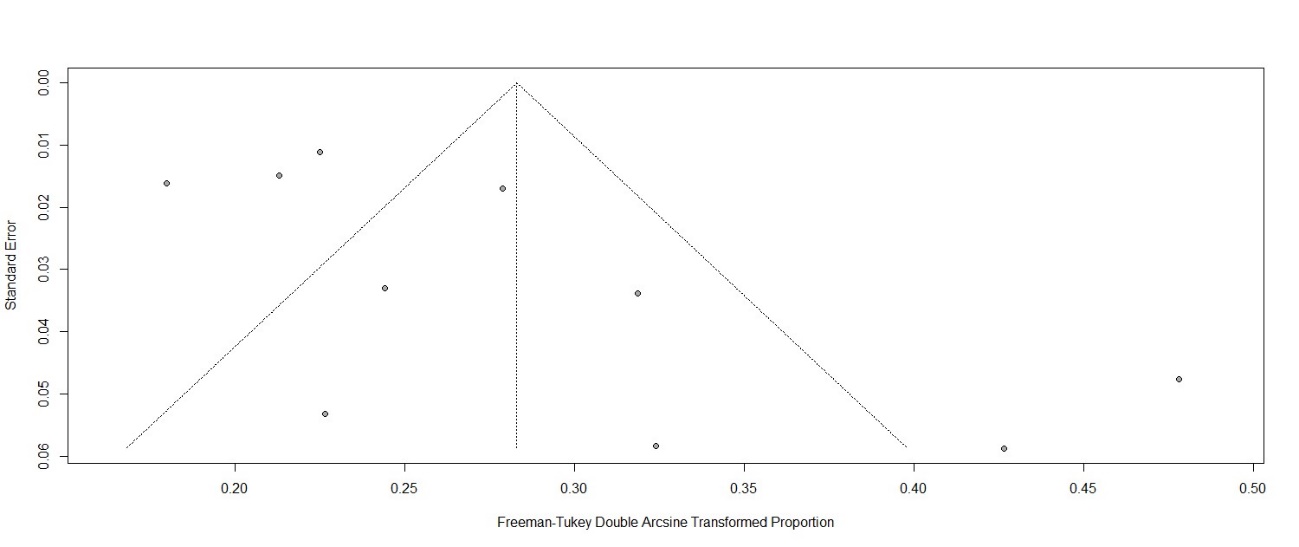


**Suppl 2** Funnel plot to test the publication bias in 10 studies with 95% Confidence limits


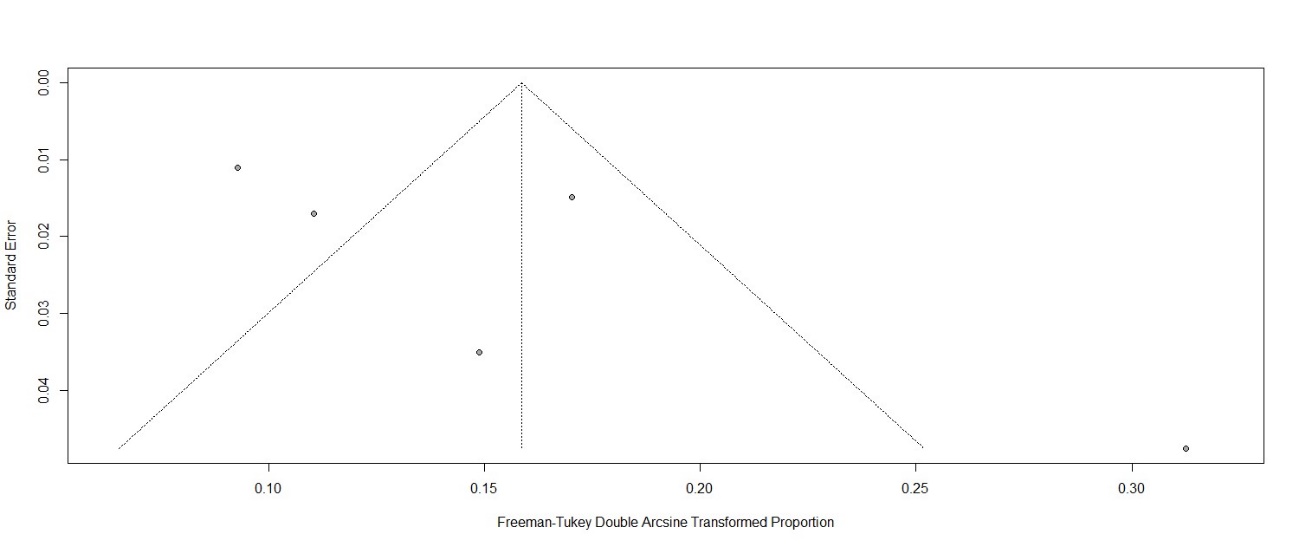


**Supp 3** Funnel plot to test the publication bias in 5 studies with 95% Confidence limits


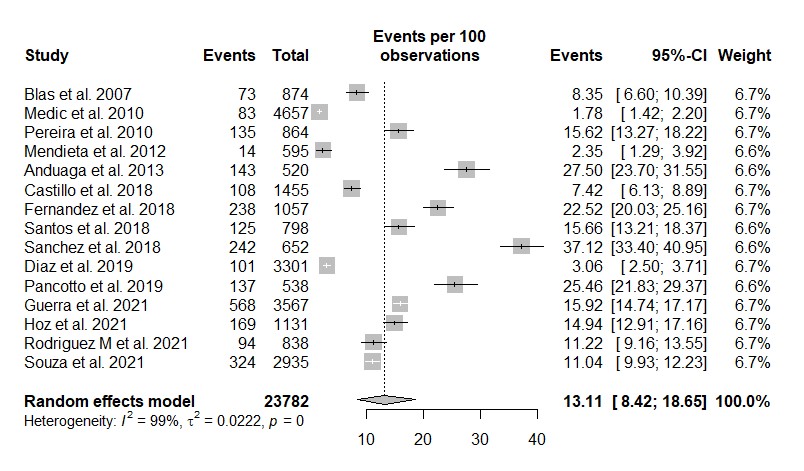


**Supp 4** Prevalence of bacteriuria in pregnant women in Latin America, considering only articles published with samples greater than 500 individuals


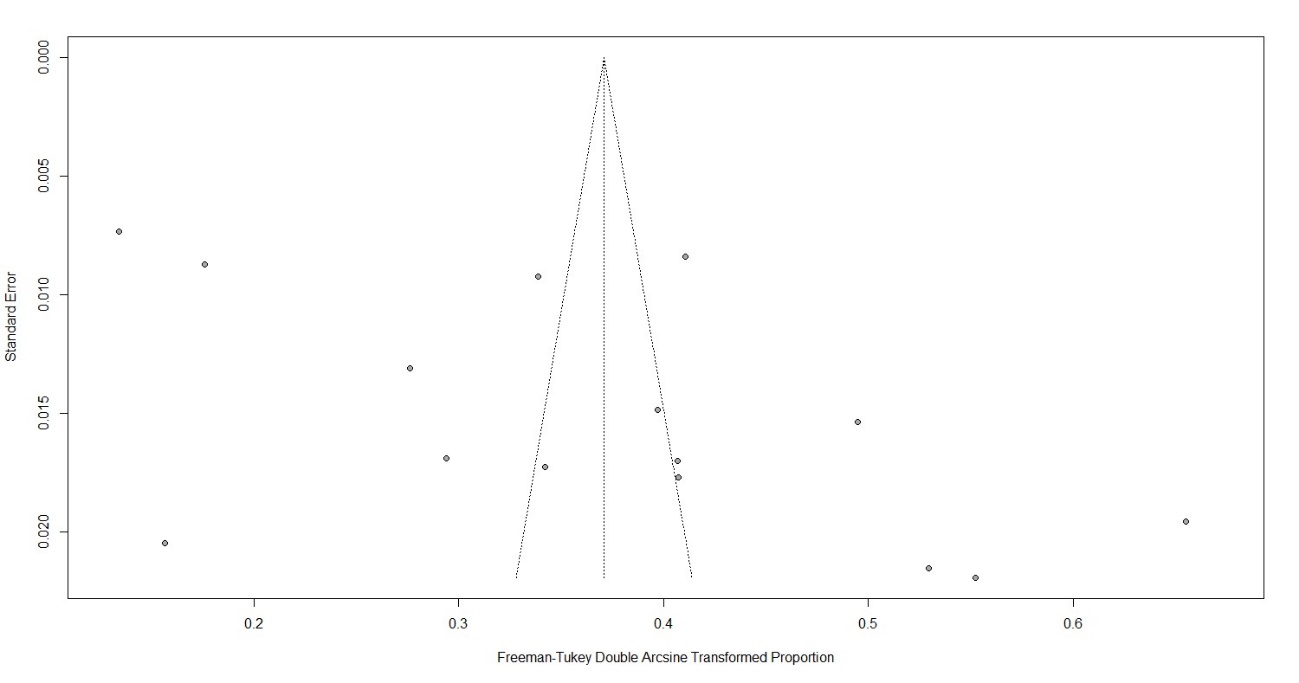


**Supp 5** Funnel plot to test the publication bias in 15 studies with 95% Confidence limits


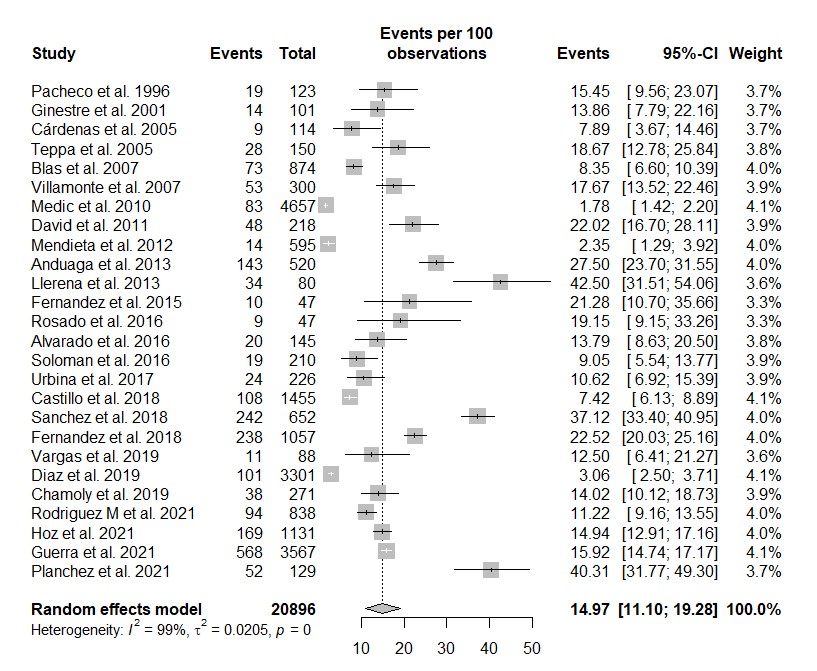


**Supp 6** Prevalence of bacteriuria in pregnant women in Latin America, with the exception of brazilian articles


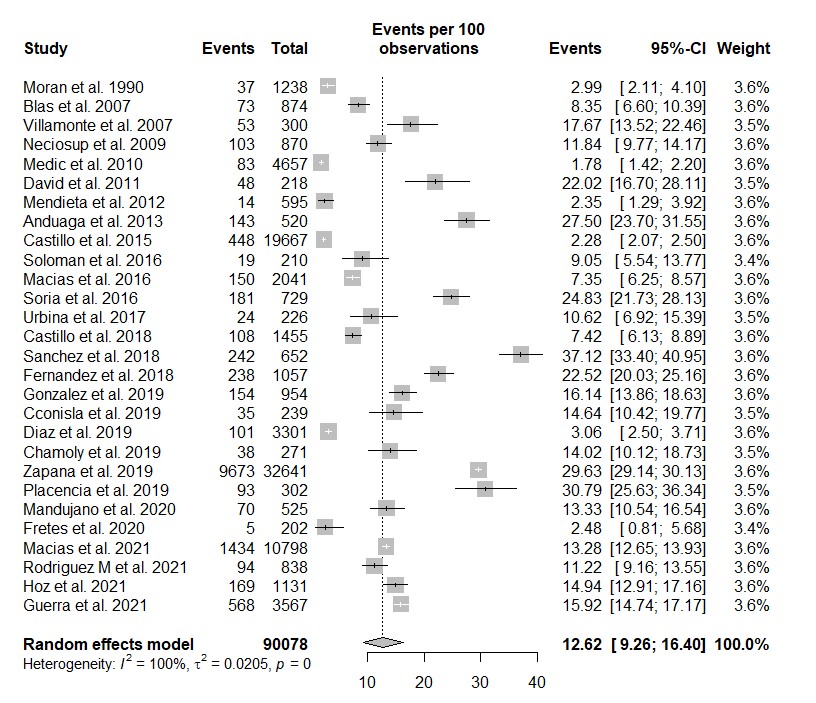


**Supp 7** Prevalence of bacteriuria in pregnant women in Latin America, with the exception of brazilian articles, in studies with samples greater than 200 individuals


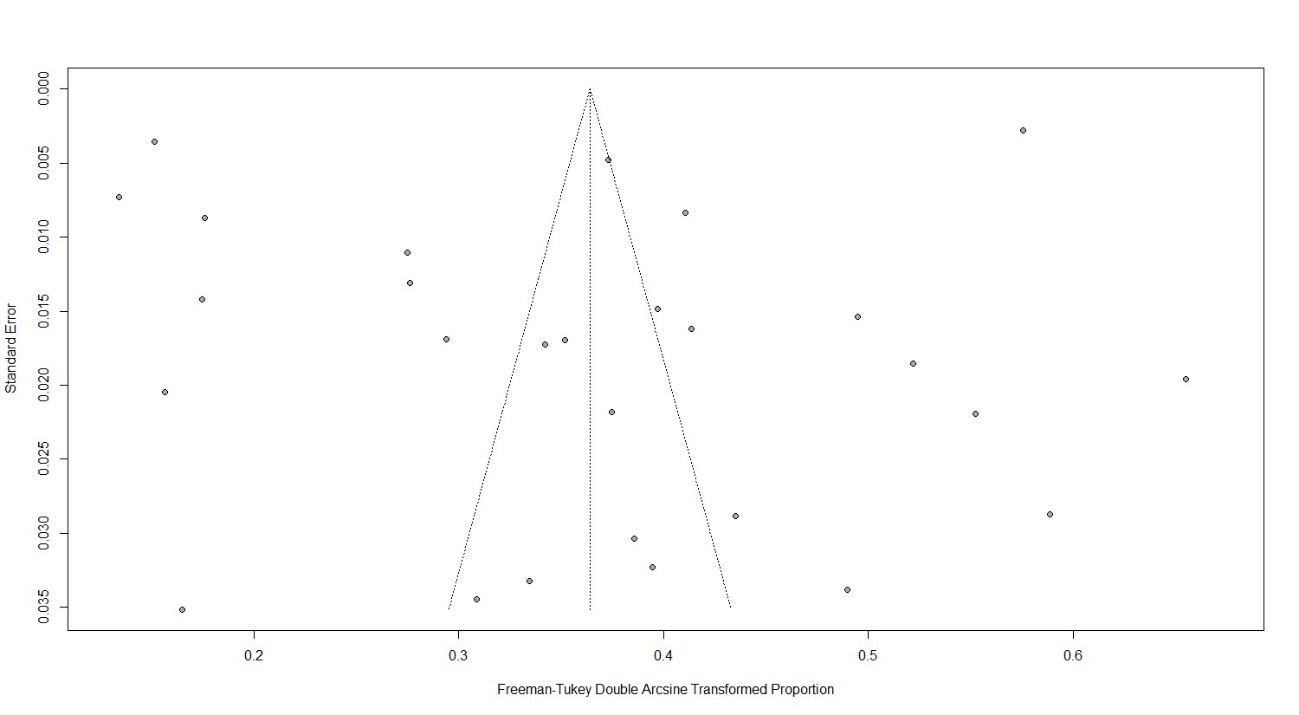


**Supp 8** Funnel plot to test the publication bias in 28 studies with 95% Confidence limits


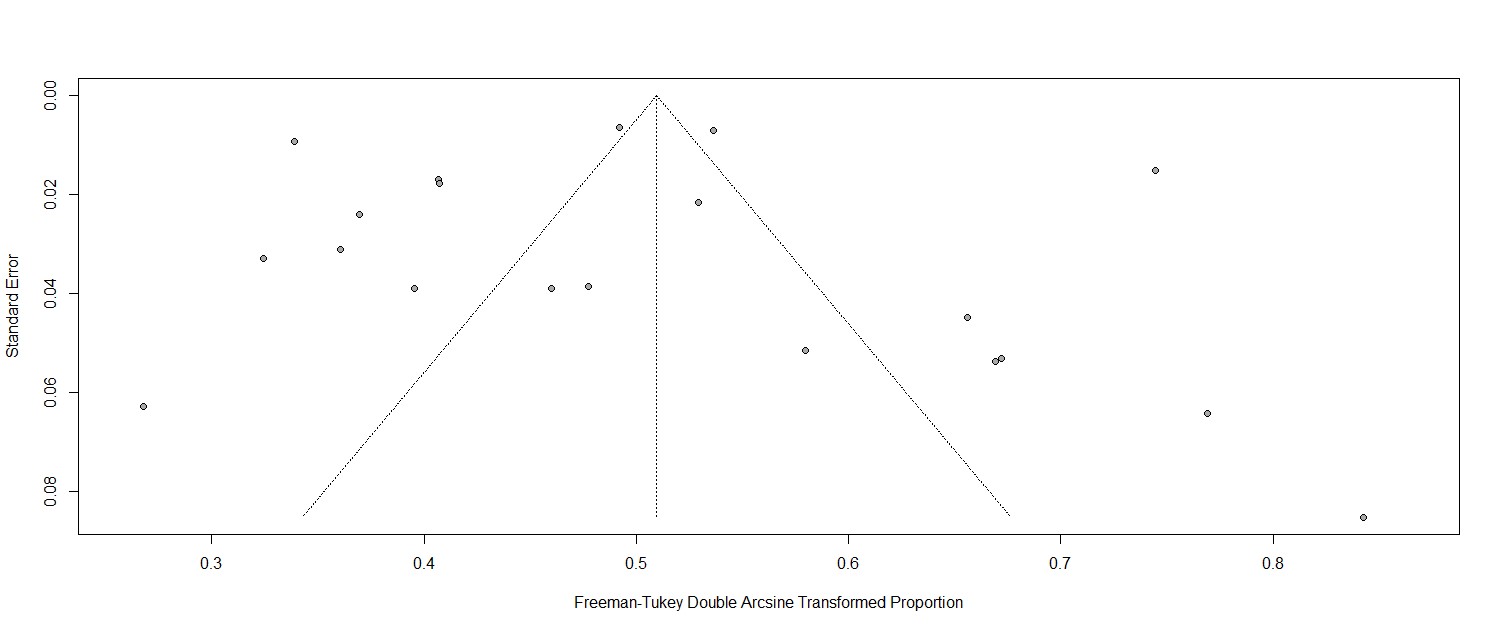


**Supp 9** Funnel plot to test the publication bias in 20 studies with 95% Confidence limits


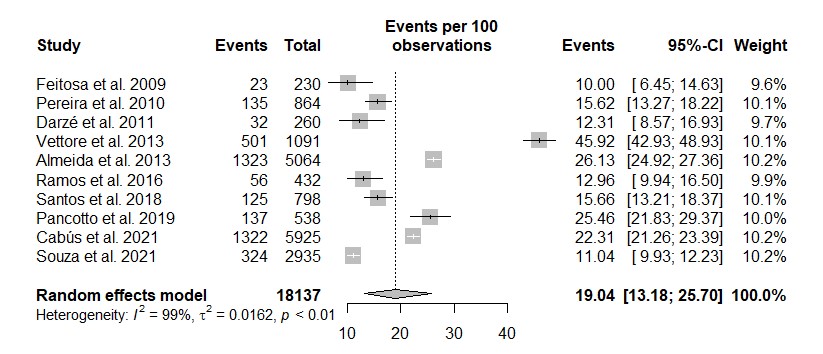


**Supp 10** Prevalence of bacteriuria in brazilian pregnant women, considering published or unpublished studies, in studies with samples greater than 200 individuals


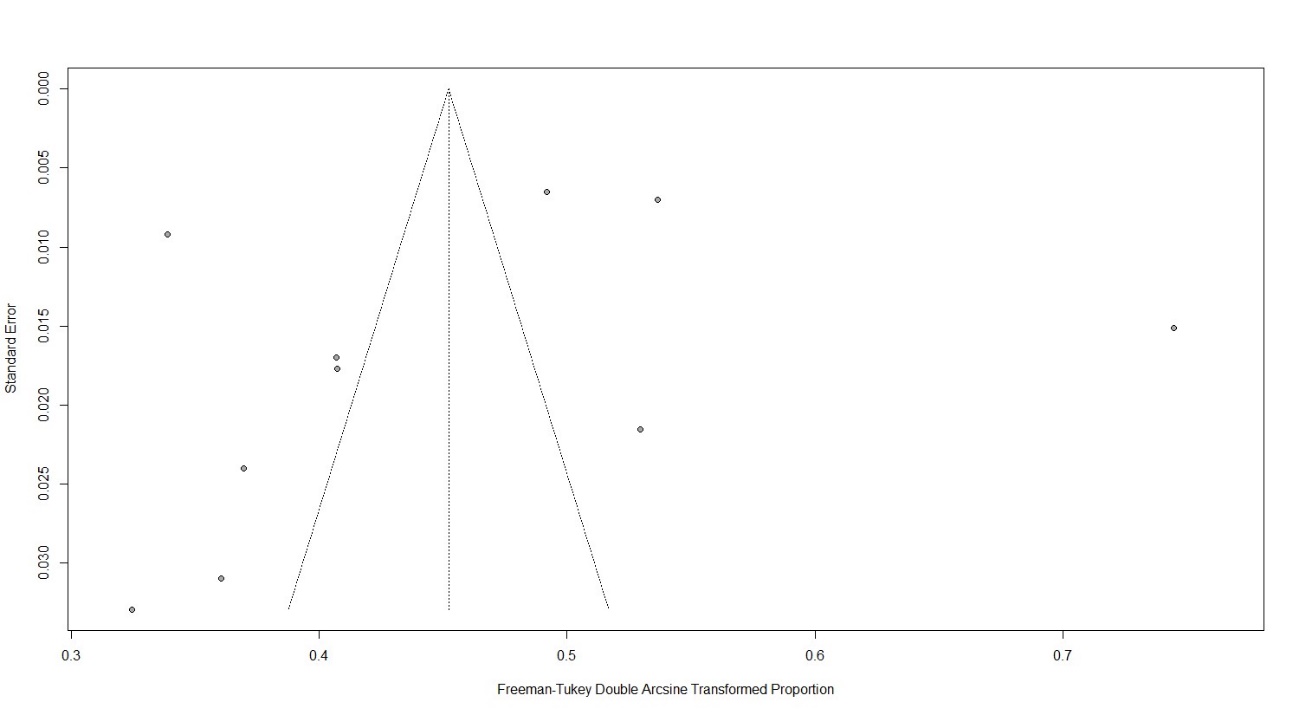


**Supp 11** Funnel plot to test the publication bias in 10 studies with 95% Confidence limits


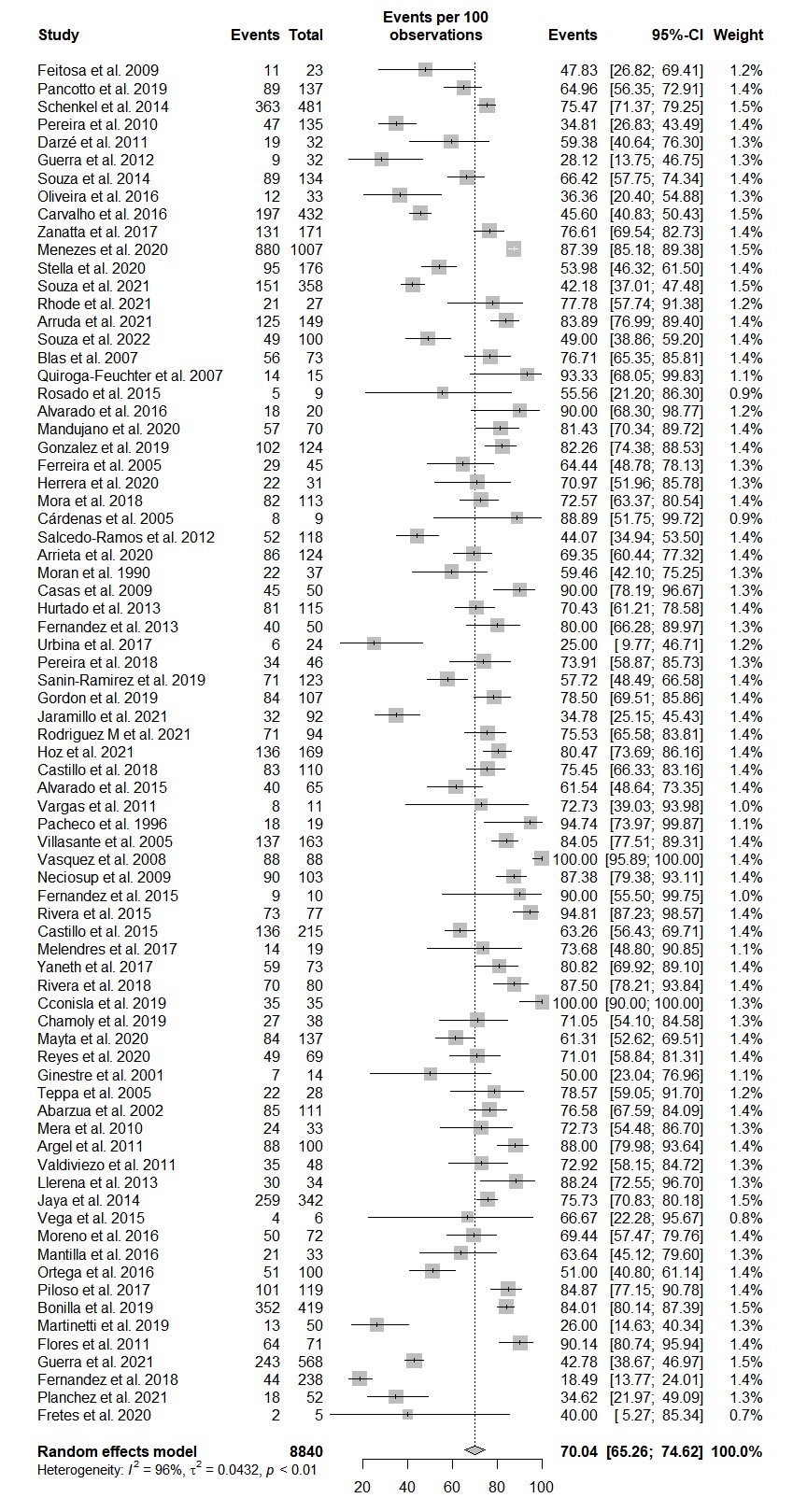


**Supp 12** Prevalence of Escherichia coli among the total number of uropathogens isolated from urine cultures of Latin American pregnant women


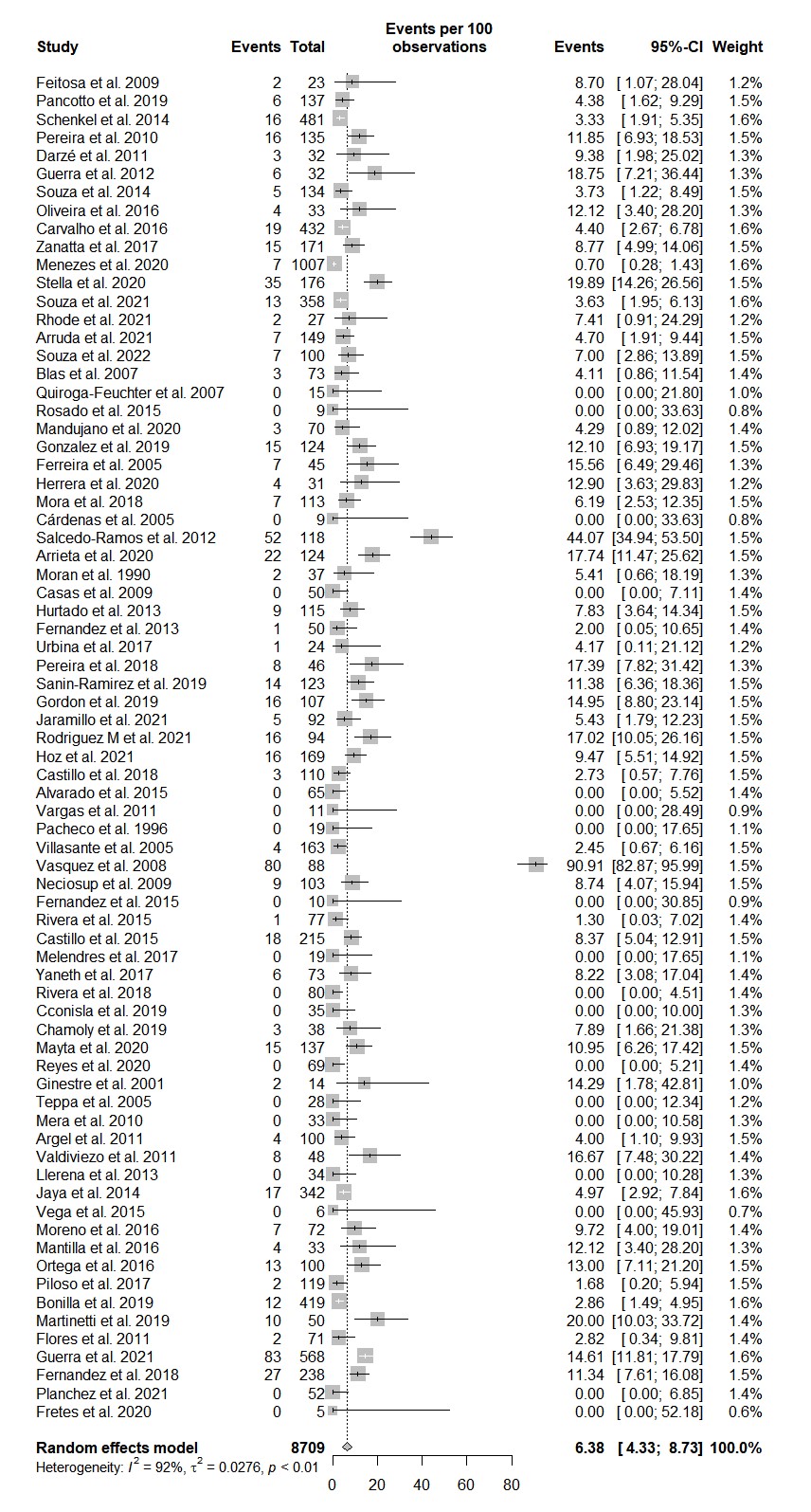


**Supp 13** Prevalence of Klebsiella sp. among the total number of uropathogens isolated from urine cultures of Latin American pregnant women


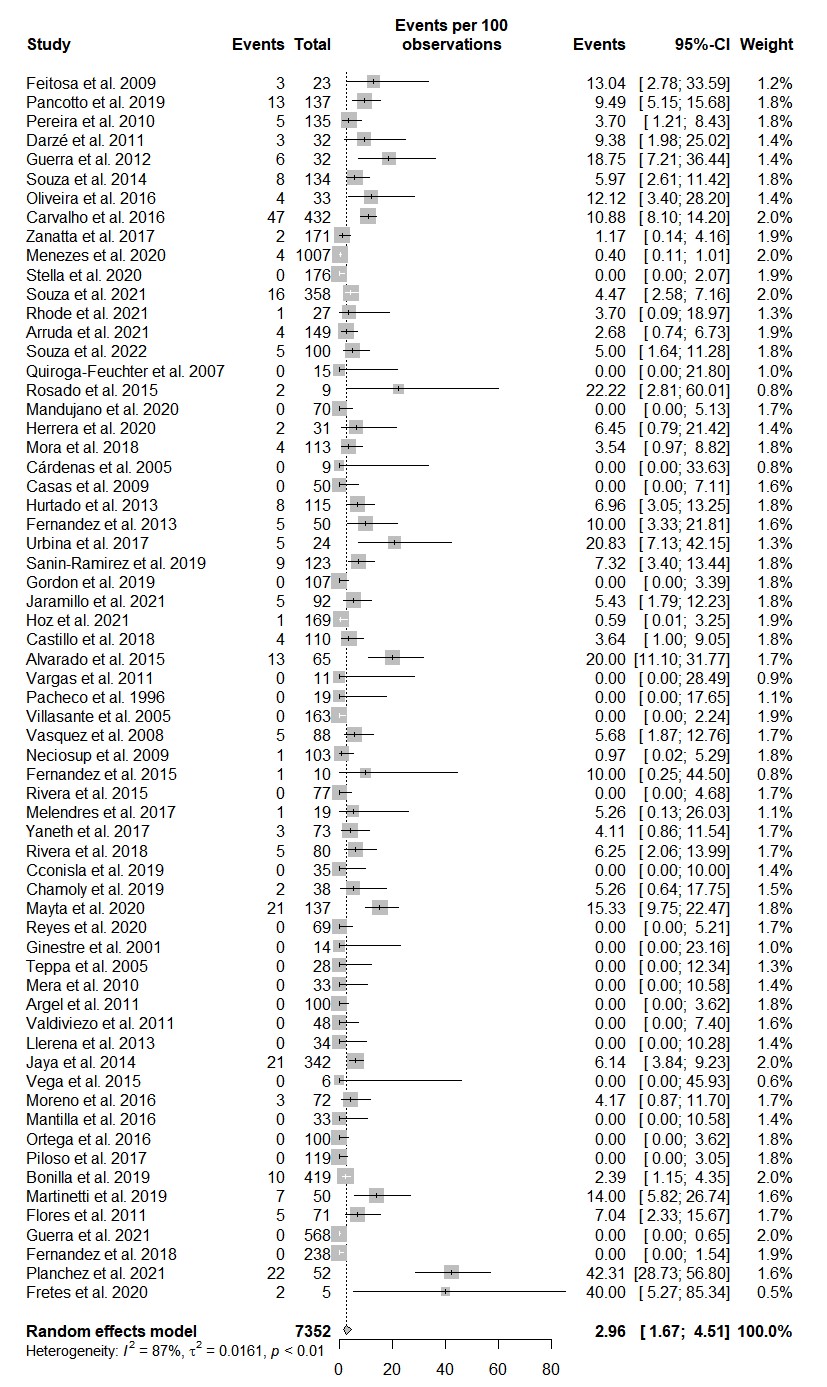


**Supp 14** Prevalence of Staphilococcus sp. (except Staphilococcus aureus) among the total number of uropathogens isolated from urine cultures of Latin American pregnant women


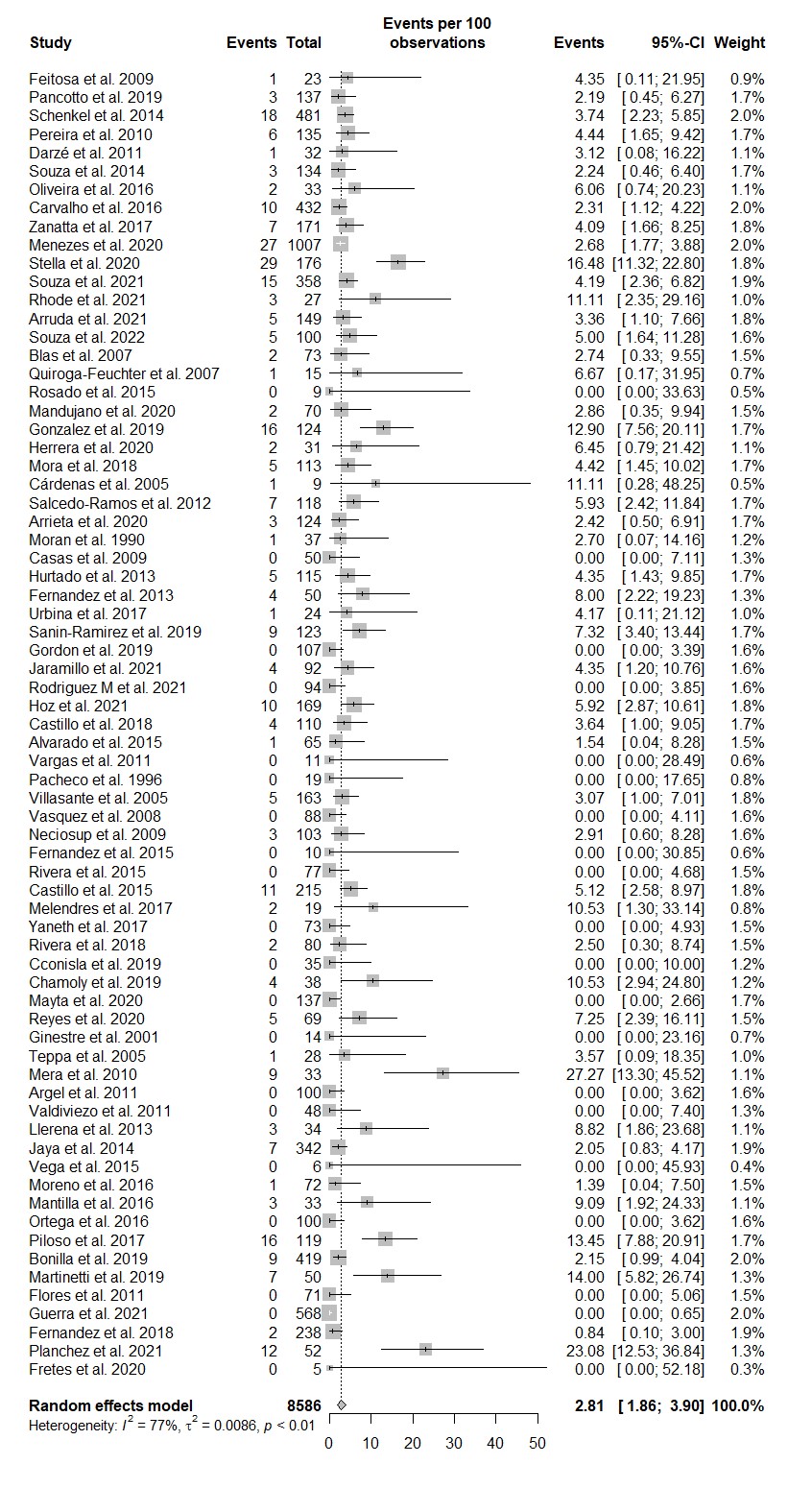


**Supp 15** Prevalence of Proteus mirabilis among the total number of uropathogens isolated from urine cultures of Latin American pregnant women


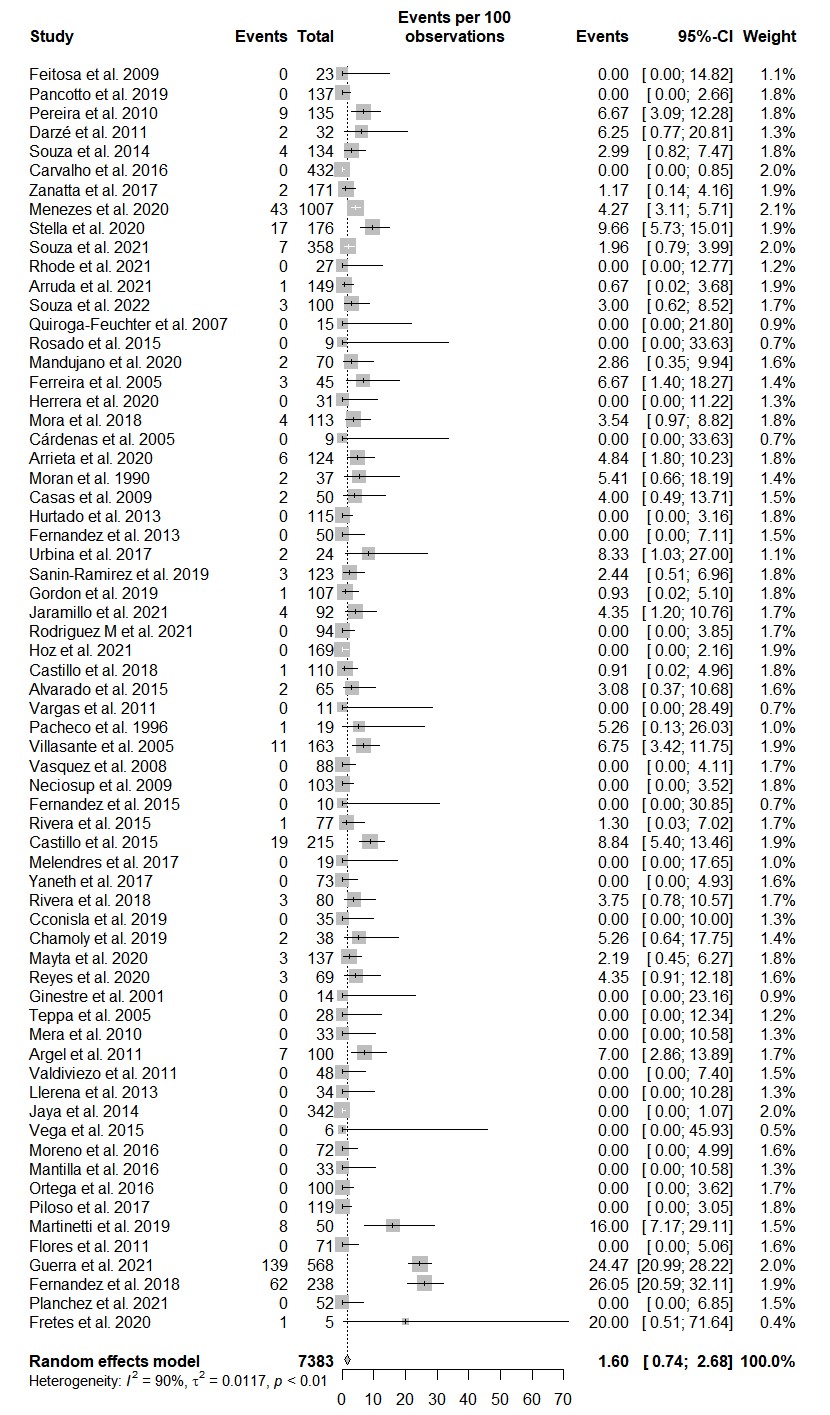


**Supp 16** Prevalence of Enterobacter sp. among the total number of uropathogens isolated from urine cultures of Latin American pregnant women


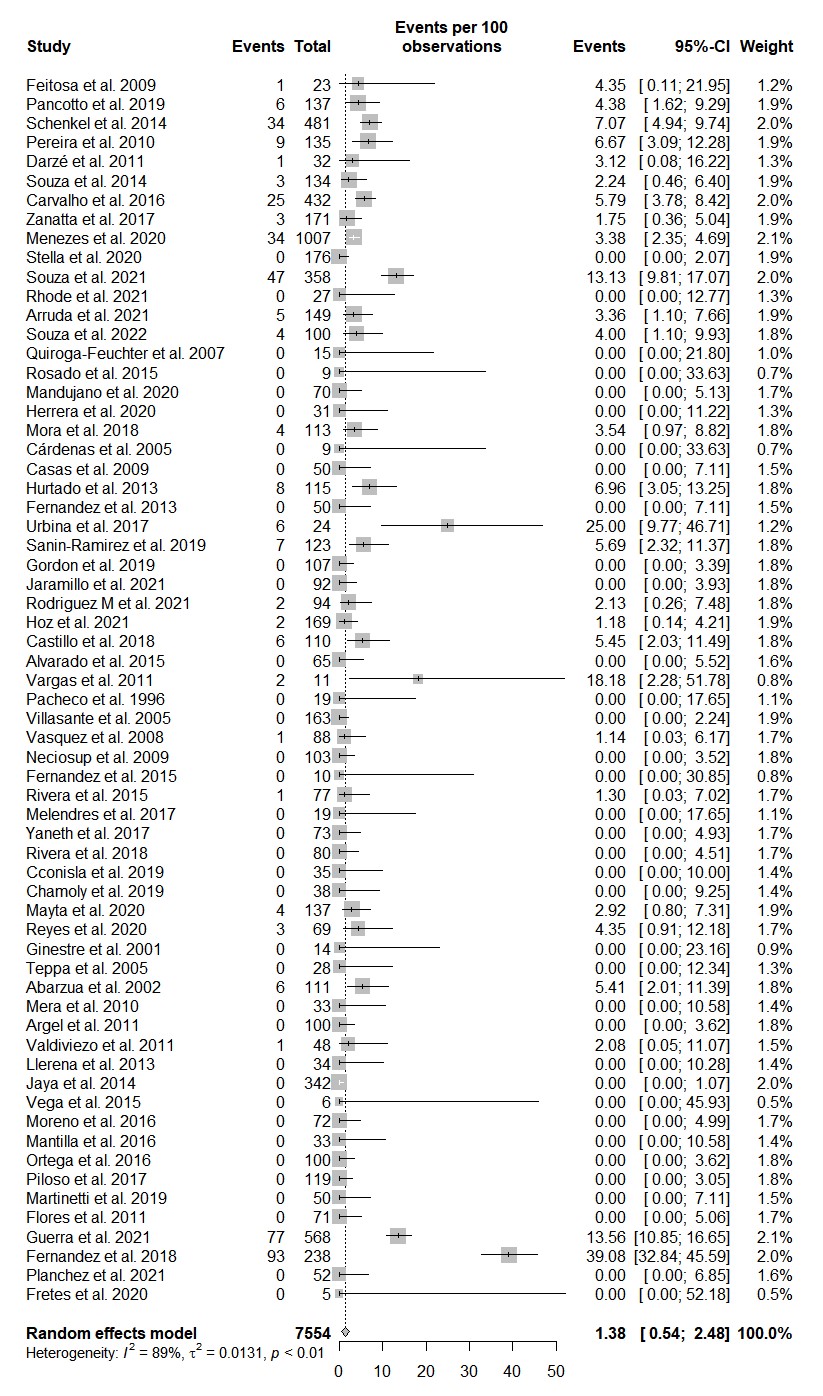


**Supp 17** Prevalence of Enterococcus sp. among the total number of uropathogens isolated from urine cultures of Latin American pregnant women


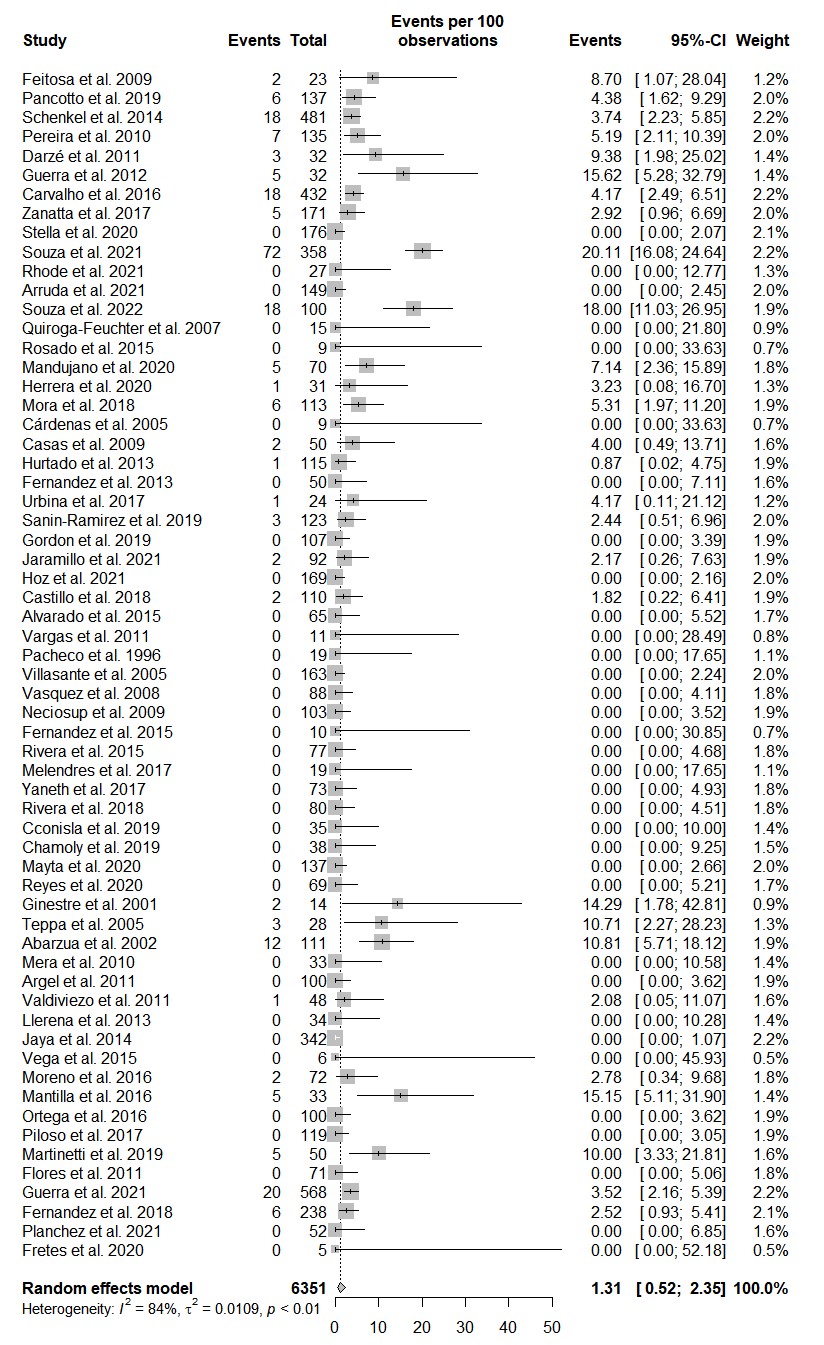


**Supp 18** Prevalence of Streptococcus agalactiae among the total number of uropathogens isolated from urine cultures of Latin American pregnant women


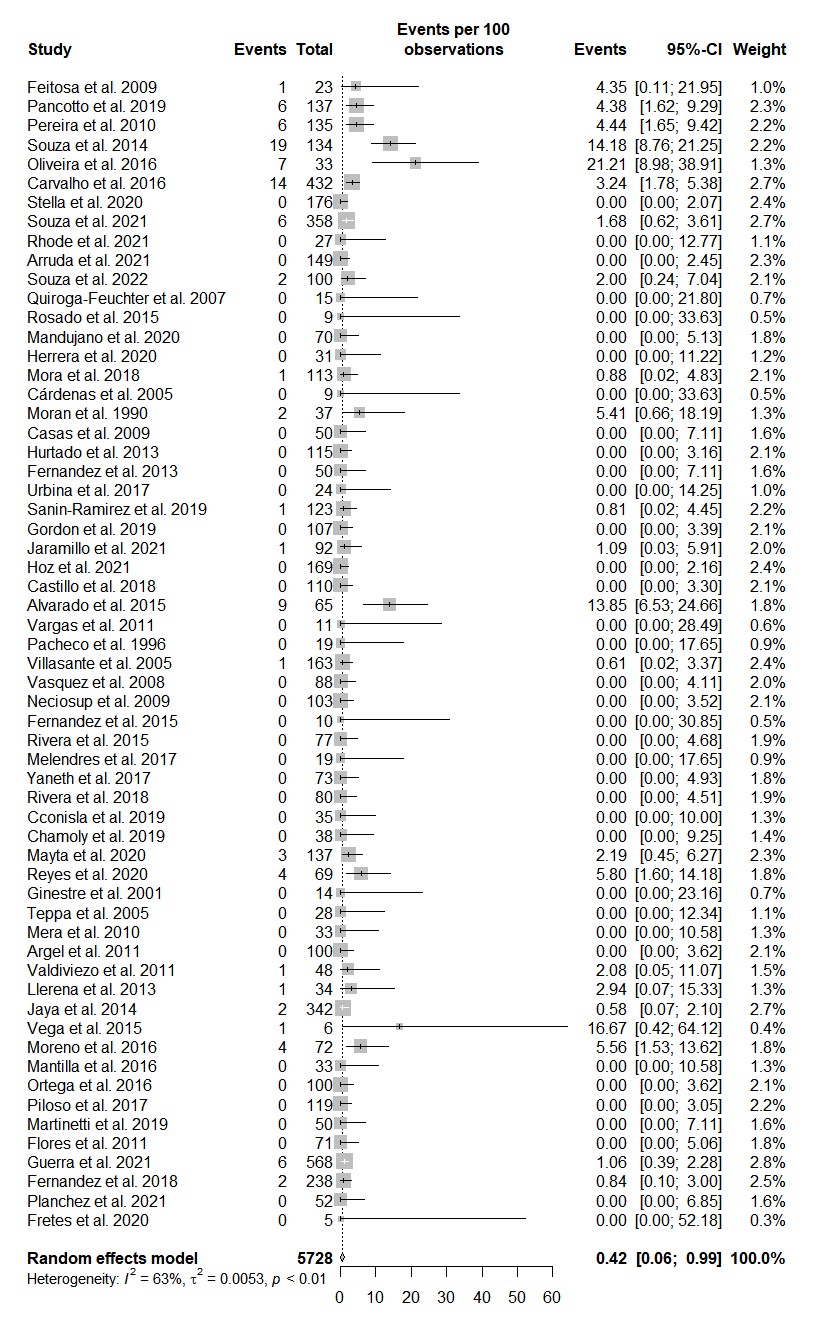


**Supp 19** Prevalence of Staphilococcus aureus among the total number of uropathogens isolated from urine cultures of Latin American pregnant women


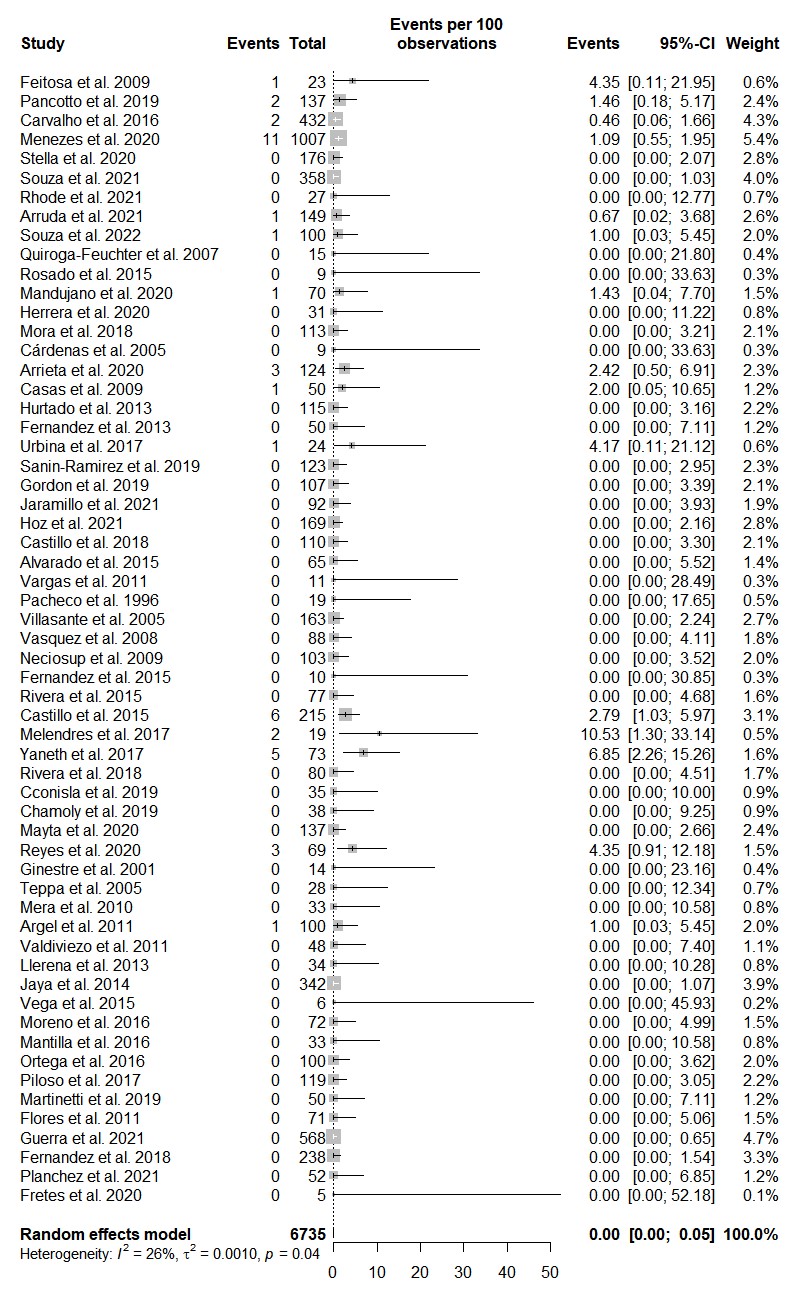


**Supp 20** Prevalence of Citrobacter sp. among the total number of uropathogens isolated from urine cultures of Latin American pregnant women


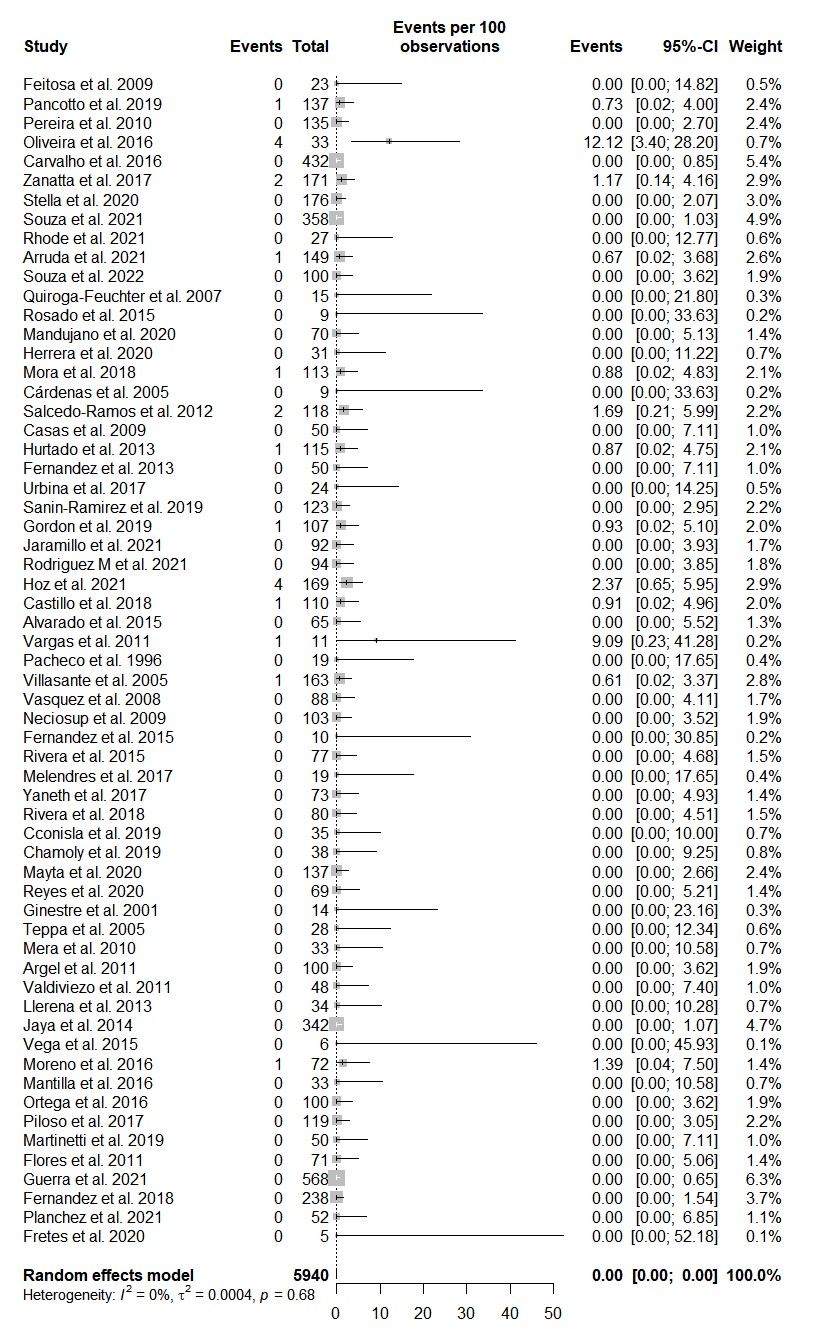


**Supp 21** Prevalence of Pseudomonas aeruginosa among the total number of uropathogens isolated from urine cultures of Latin American pregnant women
